# The molecular determinants of *R-*roscovitine block of hERG channels

**DOI:** 10.1371/journal.pone.0217733

**Published:** 2019-09-03

**Authors:** Bryan Cernuda, Christopher Thomas Fernandes, Salma Mohamed Allam, Matthew Orzillo, Gabrielle Suppa, Zuleen Chia Chang, Demosthenes Athanasopoulos, Zafir Buraei

**Affiliations:** 1 Department of Biology, Pace University, New York, NY, United States of America; 2 Department of Chemistry and Physical Sciences, Pace University, New York, NY, United States of America; Indiana University, UNITED STATES

## Abstract

Human ether-à-go-go-related gene (Kv11.1, or hERG) is a potassium channel that conducts the delayed rectifier potassium current (I_Kr_) during the repolarization phase of cardiac action potentials. hERG channels have a larger pore than other K^+^channels and can trap many unintended drugs, often resulting in acquired LQTS (aLQTS). *R*-roscovitine is a cyclin-dependent kinase (CDK) inhibitor that induces apoptosis in colorectal, breast, prostate, multiple myeloma, other cancer cell lines, and tumor xenografts, in micromolar concentrations. It is well tolerated in phase II clinical trials. *R*-roscovitine inhibits open hERG channels but does not become trapped in the pore. Two-electrode voltage clamp recordings from *Xenopus* oocytes expressing wild-type (WT) or hERG pore mutant channels (T623A, S624A, Y652A, F656A) demonstrated that compared to WT hERG, T623A, Y652A, and F656A inhibition by 200 μM *R*-roscovitine was ~ 48%, 29%, and 73% weaker, respectively. In contrast, S624A hERG was inhibited more potently than WT hERG, with a ~ 34% stronger inhibition. These findings were further supported by the IC_50_ values, which were increased for T623A, Y652A and F656A (by ~5.5, 2.75, and 42 fold respectively) and reduced 1.3 fold for the S624A mutant. Our data suggest that while T623, Y652, and F656 are critical for *R*-roscovitine-mediated inhibition, S624 may not be. Docking studies further support our findings. Thus, *R-*roscovitine’s relatively unique features, coupled with its tolerance in clinical trials, could guide future drug screens.

## Introduction

Human ether-à-go-go-related gene, or hERG [Kv11.1], is a voltage-gated potassium channel critical for nerve and cardiac function [1,2]. In the heart, hERG channels initially open during the depolarization phase of the cardiac action potential (cAP) but immediately inactivate. Upon cAP repolarization, hERG channels quickly recover from inactivation and reopen, which allows the ensuing large K^+^ efflux to speed cAP repolarization [1], limit cardiac excitability, and maintain normal QT intervals [3]. The cAP repolarization ultimately leads to hERG channel closing (deactivation). Mutations in hERG are one of the leading causes of congenital long QT syndrome (cLQTS), with a neonatal incidence rate of up to 1 in 2,500 [4]; the abnormal cardiac phenotypes are usually triggered during exercise, arousal, or rest [5].

hERG channels are tetrameric proteins, each monomer consisting of six transmembrane alpha helices (S1-S6) and cytoplasmic amino and carboxy termini [6]. Similar to other voltage-gated channels, S1-S4 is considered the primary voltage-sensing region, with S4 containing positively-charged residues that move slowly outward to induce the characteristically slow activation kinetics of hERG [2,7]. The four S5 and S6 helices and their intervening sections, which include the P-loops and P-helices, form the pore and selectivity filter of the channel [8,9]. The pore is thought to have a region between the pore helix and the S6 segments that provide pockets for drug binding [10]. Together with a strong negative electrostatic potential, the hERG pore becomes highly susceptible to unwanted drug interactions, which can result in current inhibition and acquired long QT syndrome (aLQTS) [10]. aLQTS greatly predisposes individuals to lethal cardiac arrhythmias [11], and is by far more common than cLQTS. It is estimated that 1 out of every 5 intensive care unit patients experiences a form of cardiac arrhythmia that is highly influenced by QT-prolonging medications [12–14]. Because of the dangerous side effects, pharmaceuticals have been routinely screened for hERG interaction [15,16]. Unfortunately, this has led to the loss of promising drugs that may not have posed a clinical proarrhythmic risk [17]. In fact, many clinically-safe drugs subsequently turned out to inhibit hERG, which prompted the CiPA initiative (Comprehensive in Vitro Proarhythmia Assay) that proposes the re-evaluation of previously discarded yet promising drugs; the features of such drugs are under investigation [17].

*R*-roscovitine is a cyclin-dependent kinase (CDK) inhibitor that has been used in clinical trials for cancer and for Cushing’s disease, where its effective concentration is in the ~10 μM range [18,19]. We previously found that *R-*roscovitine also blocks voltage-gated K^+^channels by binding to the open state [20]. This interaction may partially contribute to *R*-roscovitine’s anticancerogenic effects since voltage-gated K^+^channels, including hERG channels, are overexpressed in many cancer cell lines and are linked to tumorigenesis [21,22]. *R*-roscovitine inhibits hERG channels in a unique fashion [23]: 1) it blocks open hERG channels and does not become trapped in the pore during channel inactivation or closing, 2) it has low or no preference to closed and inactivated states, since inhibition of WT and the S620T inactivation deficient mutant are similar [23], and 3) it does not exhibit use-dependence, suggesting very rapid block and unblock kinetics [23]. These distinct characteristics, as well as the absence of arrhythmic side effects during clinical trials [18], alluded to a potentially unique binding mechanism for *R*-roscovitine compared to other hERG inhibitors. Thus, we set out to investigate hERG residues implicated in *R-*roscovitine-mediated inhibition.

Almost all hERG inhibitors investigated thus far are thought to bind to residues within the channel pore and that face the central cavity. These are residues that are in or near the selectivity filer (T623 and S624) and on the S6 helix (Y652 and F656) [9,24,25]. Using electrophysiological recordings on mutant hERG channels, we found that two hERG residues (S624 and Y652) that, often concordantly contribute to drug interactions [26], have opposing effects on *R*-roscovitine mediated inhibition. Specifically, hERG Y652A somewhat resisted *R*-roscovitine inhibition while hERG S624A currents were strongly inhibited, significantly more so than WT currents. We also characterized two other residues frequently involved in binding to hERG blockers: T623 and Y652. Mutation of either residue significantly weakened *R-*roscovitine block. Further analyses suggests that while some of the mutants’ altered gating properties may allosterically contribute to their attenuated inhibition by *R-*roscovitine, a more likely reason for their involvement is their binding to *R-*roscovitine, with F656 being the most critical. Further docking studies support our experimental results. These molecular determinants highlight a complexity that may guide future work on unique drugs, such as *R-*roscovitine derivatives, that exhibit open state block specificity. Our results are also the first to shed light on the residues involved in hERG inhibition by a trisubstituted purine, which is important as many *R-*roscovitine derivatives are now being developed as anti-cancer treatments [27,28].

## Methods

### RNA preparation and oocyte injection

WT and mutant (T623A, S624A, Y652A, and F656A) hERG1a clones, which were subcloned into a pSP64 vector, were ordered from Addgene (catalog #53051 for WT, #53055 for T623A, #53056 for S624A, #53053 for Y652A, and #53054 for F656A). The vectors were linearized with EcoRI and *in vitro* transcribed using the mMESSAGE mMACHINE SP6 Transcription Kit (Ambion). 50 nl of the resulting cRNA was injected (using Nanoject II and glass capillaries, both from Drummond Scientific) into defolliculated *Xenopus laevis* oocytes. There were two sources for the oocytes: 1) they were extracted from frogs purchased from Xenopus 1 (Michigan), using IACUC-approved procedures at Columbia University and generously gifted to us (by Dr. Jian Yang lab); and 2) we purchased *Xenopus laevis* ovaries from Xenopus 1, and extracted stage V-VI oocytes as previously described [29]. Briefly, 2–3 mm size strips cut from *Xenopus laevis* ovaries were treated for ~ 1.5 hours (shaking at 175 rpm, room temperature) with a solution containing 0.3–0.5 mg/ml collagenase A (Boehringer Mannheim), 82.5 mM NaCl, 2.5 mM KCl, 1 mM MgCl_2_, and 5 mM HEPES (pH 7.6). A 15-minute rinse on a shaking incubator set to 75 rpm was repeated twice with the ND96 solution that contained 96 mM NaCl, 2.5 mM KCl, 1 mM MgCl_2_, 5 mM HEPES, 1.8 mM CaCl_2_, 100 units/mL penicillin, 100 μg/mL streptomycin (pH 7.6), and 5 mM sodium pyruvate. Following the selection of defoliculated oocytes, hERG cRNAs were injected into the oocytes and recordings were performed 2–7 days later. All procedures were in accordance with regulations set by the institutional animal care and use committee.

### Solutions and drug administration

Recording electrodes were pulled on a Narishige PC-10 puller from Clark capillary glass (#30–0038, Harvard Apparatus) and used with a resistance of 1–5 MΩ. The intracellular electrode solution contained 3M KCl, while the low potassium extracellular solution (low K^+^) contained [in mM]: 5 KCl, 100 NaCl, 1.5 CaCl_2_, 2 MgCl_2_, and 10 HEPES. To elicit large tail currents from hERG pore mutants with reduced permeation (F656 and T623) we used a high K^+^ extracellular solution containing [in mM]: 96 KCl, 2 NaCl, 1.8 CaCl_2_, 1 MgCl_2_, 5 HEPES. This had been established as a necessary procedure to overcome the very small currents, reduced expression, altered permeation, and strong inward rectification found in the F656A and T623A pore mutants [30–34]. The pH of the external solutions were adjusted to 7.4 using either 1M NaOH for the low K^+^, or 1M KOH for the high K^+^ solution, and the osmolarities were ~200 mOsm. *R*-roscovitine was dissolved in DMSO to make a 100 mM stock solution that was frozen at -80°C. The solubility of *R-*roscovitine in DMSO is 5 mg/ml and the maximum DMSO concentration used in our working solutions was 1%, which was used for the highest applied drug concentration of 1 mM. This concentration of DMSO is well tolerated by frog oocytes (e.g. see [35] or [36]). Drug-free external solutions (control and wash) had the same DMSO concentrations as the *R*-roscovitine-containing solutions. Working solutions were prepared on the day of the recordings.

### Two-Electrode Voltage Clamp (TEVC) Recordings

Currents were amplified using the OCT clamp TEVC instrument and data was acquired and analyzed using Clampex and Clampfit, respectively (Axon Instruments). Currents were digitized using a digidata 1440A board at 10 kHz. Leak subtraction was not applied during the recordings. Before recordings began, a pulse protocol was applied to allow hERG runup or rundown to subside. The pulse protocol consisted of a -80 mV holding potential, a 1-second depolarization to +40 mV, and a 1-second repolarization to either -50 mV (for low K^+^ external solutions) or -120 mV (for high K^+^ external solutions). The high K^+^ solution was necessary for the F656 and T623 mutants that are known to have very small currents. Because of this, the step repolarization protocol also requires an alteration to increase the driving force to -120 mV to obtain larger tail currents for analyses [30–34]. The protocol was applied for ~ 3.5 minutes. Following this period, other voltage protocols (described below) were applied. All traces were baseline-adjusted to remove the minuscule current that deviated from 0 μA at the -80 mV holding potential.

### Time-Course protocol and IC_50_ calculations

Cells were held at -80 mV. For low K^+^ outward-current hERG constructs (Y652A and S624A), a 1-second depolarizing step from -80 mV to +40 mV was followed by a 1-second repolarization to -50 mV. This pulse was applied repetitively over a span of ~ 33 minutes. A similar protocol was applied to inward-current hERG constructs (F656A and T623A), but the 1-second repolarization voltage was instead set to -120 mV. Tail currents were measured to generate a time-course of inhibition for each cell before and during application of various *R*-roscovitine concentrations: 10, 30, 100, 300, and 1000 μM. Fractional block of tail current was calculated for each *R*-roscovitine concentration as: (*I*_control_-*I*_Rosc_)/*I*_control_, with *I*_control_ being an average of the tail current before a particular *R*-roscovitine concentration and the maximum drug-free current elicited from the cell [i.e. (*I*_before_+ *I*_maxbefore_)/2]. The fractional block was then plotted against *R*-roscovitine concentrations and fitted with the following Hill equation to obtain IC_50_ values: Y = Bottom+(Top-Bottom)/(1+10^((LogIC_50_-X)*HillSlope)), where X is the log of the concentration, Top and Bottom (constrained to 1 and 0, respectively) are the curve plateaus, LogIC_50_ is the center of curve, and HillSlope is the slope factor.

### Step depolarization and step repolarization protocols

To generate step I-V curves, cells were held at -80 mV and 2-second voltage steps were applied in 10 mV increments from -50 mV to +60 mV. The resulting step currents were measured as current means from the final 50 ms of the depolarization, which were then normalized and plotted against step voltages. Following the depolarizing steps, cells were repolarized to -50 mV for 1.5 seconds, eliciting tail currents. Peak tail currents were normalized and plotted against step voltages for tail I-V curves, which were fitted with the following Boltzmann sigmoidal equation to obtain activation curves: Y = Bottom+(Top-Bottom)/(1+exp((V_50_-X)/Slope)), where Top and Bottom are the curve plateaus (constrained to 1 and 0, respectively), V_50_ is the step voltage that elicits 50% channel activation, and Slope is the steepness of the curve. Percent inhibition of step and tail currents with 200 μM *R*-roscovitine was calculated as: (*I*_control_-*I*_Rosc,200_μ_M_)/*I*_control_, with *I*_control_ being the current size before 200 μM *R*-roscovitine application.

A step repolarization protocol was used for inward-current hERG constructs. Following 1-second depolarization steps to +60 mV, 2-second repolarization voltages were applied in 20 mV decrements from +20 mV to -120 mV. The resulting peak tail currents were then normalized and plotted against tail voltages to acquire tail I-V curves, which were fitted with a second-order polynomial equation (quadratic): Y = B0+B1*X+B2*X^2^. To obtain reversal potentials (*E*_rev_), the equation was solved for Y = 0. Similar to step and tail current inhibitions [during step depolarization protocols] with 200 μM *R*-roscovitine, percent inhibition of tail currents with 500 μM *R*-roscovitine was calculated as: (*I*_control_-*I*_Rosc,500_μ_M_)/*I*_control_, with *I*_control_ being the current size before 500 μM *R-*roscovitine application. To obtain time constants of deactivation, we used biexponential fits to the deactivation phase (tail currents) [25]. The fast component of deactivation (τ_fast_) was used for comparisons since most channels deactivate with the faster time constant, at the hyperpolarized voltages we used [25]. The following standard biexponential equation was used for fitting: $f(t)=\sum_{i=1}^{n}A_{i}e^{‐t/\tau_{i}}+C$.

### Molecular docking and graphics

The structure of an open WT hERG channel was previously generated as a homology model [37] based on the KvAP crystal structure [38]. Geometry optimization of *R*-roscovitine was achieved using the Hartree-Fock/STO3G* method in Gaussian 09 [39] and the protonation state of *R*-roscovitine in pH 7.3 was calculated using Avogadro [40]. Ligand docking with Autodock Vina [41] in PyRx [42] docked *R*-roscovitine in a gridbox that encompassed the entire S5-S6 region. Only the exhaustiveness setting was changed from default: exhaustiveness was set to 100 [43]. Information on additional docking carried out with *R-*roscovitine and the cryo-EM structure can be found in the Supporting Information. Molecular graphics was performed with the UCSF Chimera package [44]. Chimera is developed by the Resource for Biocomputing, Visualization, and Informatics at the University of California, San Francisco.

### Data analysis and statistics

Clampex was used for data acquisition and Clampfit and Microsoft Excel were used for initial data analyses. GraphPad Prism version 8.0.2 for Macintosh (GraphPad Software, La Jolla California USA) was used to make I-V, time-course, and percent inhibition graphs; it also calculated the nonlinear fits of the tail I-V and fractional block graphs (i.e. Boltzmann, four parameter logistic, and quadratic). Data are represented as mean ± standard error. All statistical analyses were performed using GraphPad Prism. Ordinary one-way ANOVA tests and Kruskal-Wallis tests were followed by post-hoc analyses: Dunnett’s multiple comparison test [for one-way ANOVAs] and Dunn’s test [for Kruskal-Wallis tests], with multiple comparisons reported as multiplicity adjusted p values. Statistical significance was designated when p < 0.05 and all p-values were subsequently reported either in the figure legends or results.

## Results

### *R*-roscovitine-mediated inhibition of WT hERG in *Xenopus* oocytes

**Fig 1:**
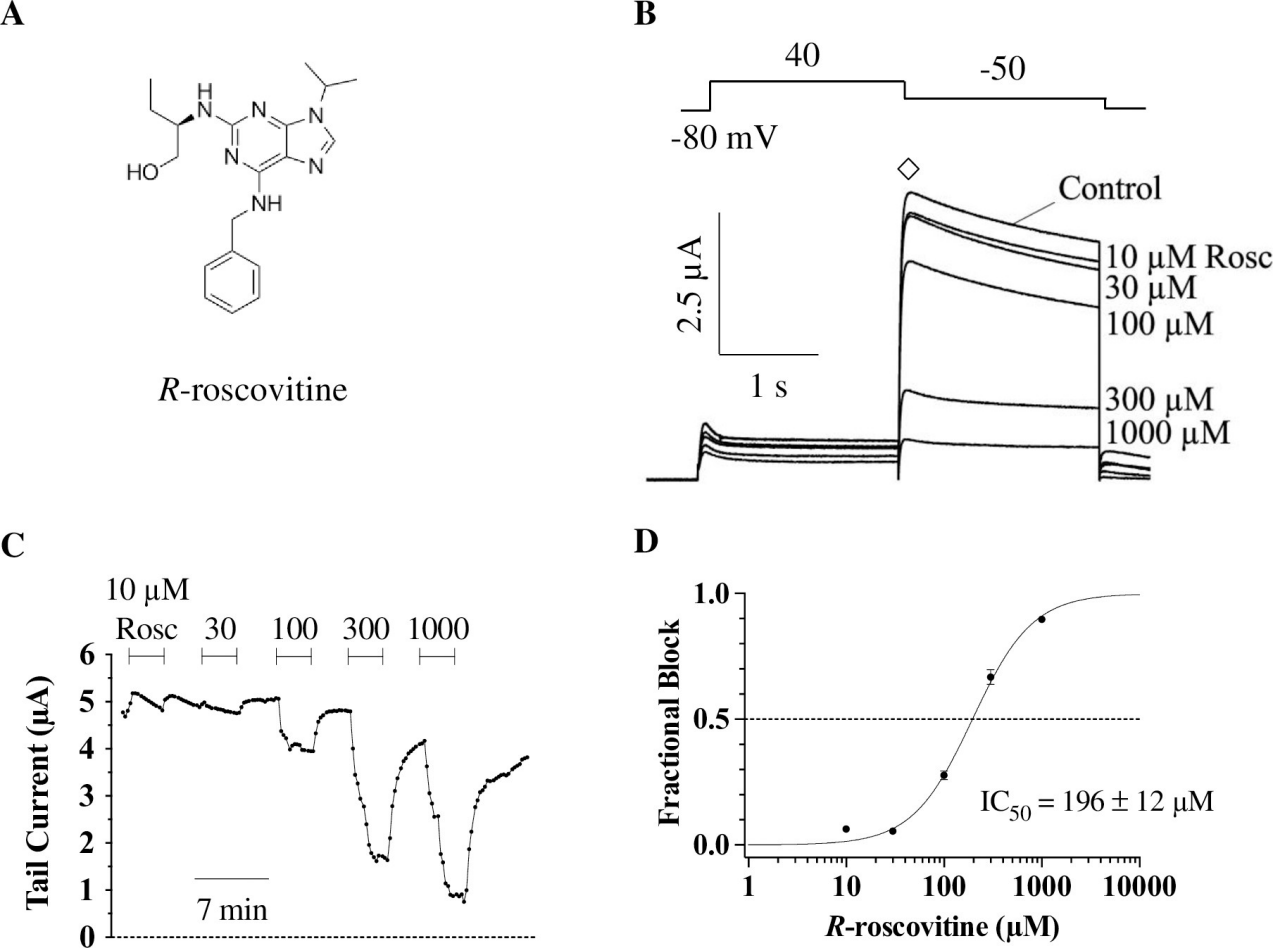
Dose-response curve for *R-*roscovitine inhibition of WT hERG channels. **A)** Skeletal formula of *R-*roscovitine, 2-(1-ethyl-2-hydroxyethylamino)-6-benzylamino-9-isopropylpurine. **B)** Pulse protocol (top) along with representative traces from a WT cell (bottom) exposed to the indicated *R-*roscovitine concentrations. Channels were activated with a 1-second step to +40 mV from -80 mV, and tail currents (◊) were elicited by a 1-second repolarization to -50 mV. The single 2-second episodic pulse was repeated for 3.44 minutes as the various concentrations were washed in and out of the gravity-fed perfusion system. **C)** Time-course of tail current inhibition (measured from ◊, in B) from a representative cell expressing WT hERG in the presence of the indicated *R-*roscovitine concentrations. **D)** Fractional block data were fitted with a Hill equation (see methods) to obtain the IC_50_ for WT hERG (196 ± 12 μM, n = 11). Error bars here and elsewhere represent standard errors.

The half-maximal inhibitory concentration (IC_50_) of *R-*roscovitine inhibition of WT hERG tail currents was previously determined to be 27 μM in HEK-293 cells [23], but *Xenopus* oocytes are known to require higher drug doses [45–47]. To determine the effectiveness of *R*-roscovitine inhibition (see *R*-roscovitine structure in Fig 1A) on WT hERG channels in *Xenopus* oocytes, we injected the oocytes with cRNA for WT hERG and recorded potassium currents 2–7 days later (see methods). The currents were elicited using a 2-second pulse protocol consisting of a 1-second depolarization to +40 mV followed by a 1-second repolarization to -50 mV, during which tail currents were measured (Fig 1B). This pulse protocol was repeated either every 5 seconds for *R-*roscovitine concentrations applied in a randomized order (100, 300, 30, and 10 μM) or every 12 seconds for ascending *R-*roscovitine concentrations (10, 30, 100, 300, and 1000 μM), with the various drug concentrations being repeatedly applied and washed off (Fig 1C). Fitting the fractional block of hERG tail currents with a Hill equation resulted in an IC_50_ for WT of 196 ± 12 μM (Fig 1D) and a slope of 1.5 ± 0.1. The 7.4-fold higher IC_50_ we found in oocytes compared to HEK cells (27 μM; [23]) was quite expected as many comparative studies typically find a 10–30 fold higher IC_50_ in *Xenopus* oocytes compared to mammalian cells [45–47]. This is thought to occur because of the vitelline membrane barrier that surrounds oocytes, as well as their yolk that acts a sink for drugs [45]. Thus, we used 200 μM *R*-roscovitine for studying inhibition levels of WT and mutant hERG channels.

**Fig 2:**
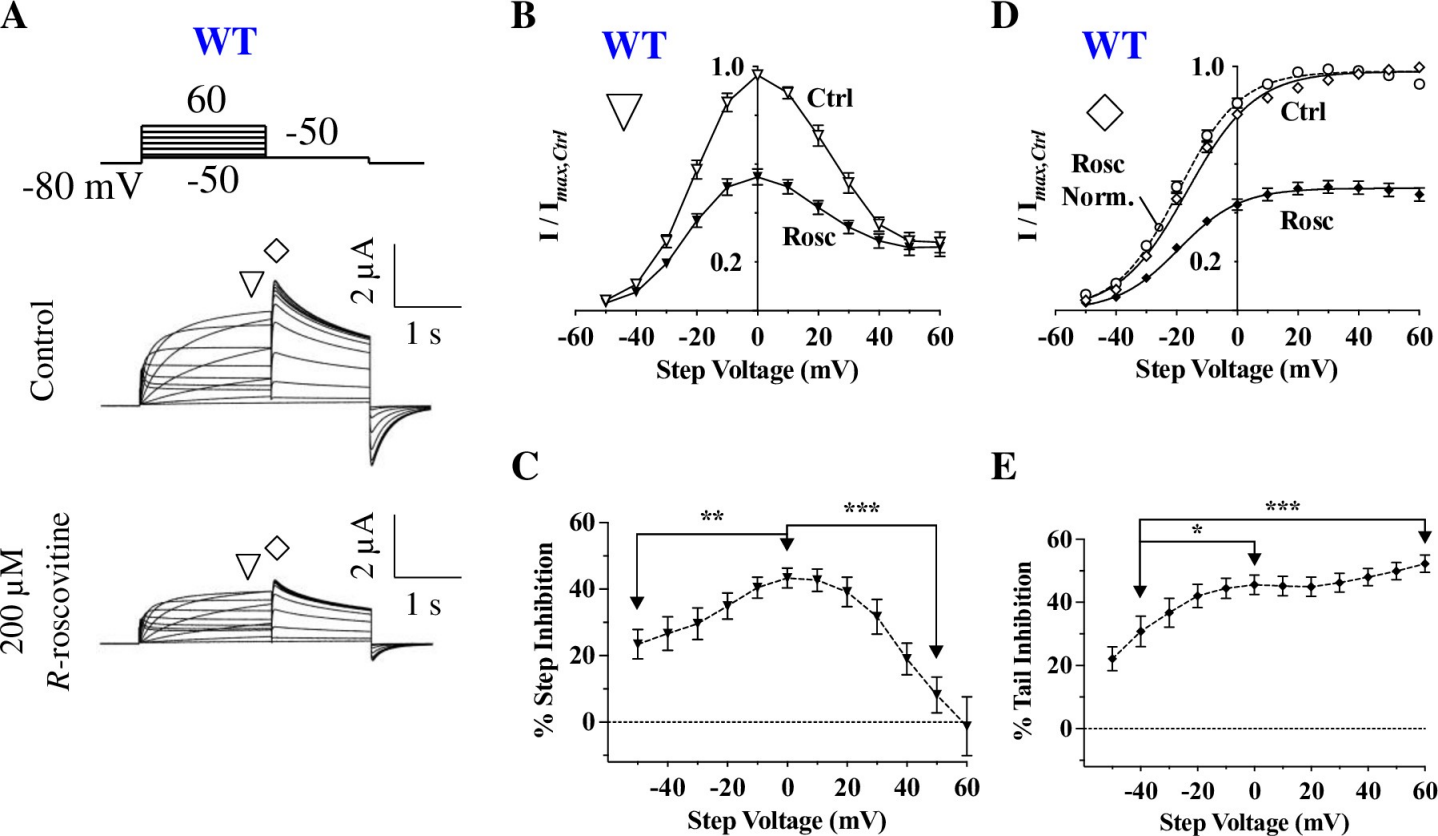
Characteristics of *R*-roscovitine inhibition of hERG channels. **A)** Voltage protocol and current traces from a representative cell expressing hERG channels before (Control) and during 200 μM *R-*roscovitine application. From a holding potential of -80 mV, outward currents were elicited at two phases: depolarization (from -50 mV to +60 mV) and repolarization (to -50 mV). Mean step currents were measured at the end of the 2-second step (▽) and tail currents were measured from their peak (◊). **B)** Normalized I-V curves for Control and 200 μM *R-*roscovitine from mean step currents (▽, n = 14). Inhibition was strongest between -30 mV and +30 mV, a range where the open state is more prevalent than either the closed or inactivated state. **C)** Average percent step current inhibition by 200 μM *R-*roscovitine at various step voltages. Inhibition was weakest at voltages where channels are closed (-50 mV) or inactivated (+50 mV; p = 0.0052 for 0 vs. -50 mV, p < 0.0001 for 0 vs. +50 mV, n = 14, one-way ANOVA). **D)** Tail I-V curves for Control and 200 μM *R-*roscovitine, with normalized peak tail currents (◊, n = 14). Smooth lines are Boltzmann fits to the activation curves, which produced significantly different V_0.5_ (Ctrl: -16.5 ± 1.1 mV, Rosc: -19.4 ± 1.1 mV; p = 0.0105, n = 14, paired *t-*test) and slopes (Ctrl: 11.7 ± 0.5, Rosc: 10.6 ± 0.5; p = 0.0114; n = 14, paired *t*-test). The dashed line shows a scaled activation curve in the presence of *R-*roscovitine. **E)** Average percent tail current inhibition by 200 μM *R-*roscovitine at various step voltages. The inhibition of tail current significantly increased with rising levels of depolarized potentials (p = 0.0134 for -40 vs. 0 mV, p = 0.0004 for -40 vs. +60 mV, n = 14, one-way ANOVA). Error bars represent standard errors; when not visible, error bars are smaller than the symbols. * = *P* < 0.05, ** = *P* < 0.01, and *** = *P* < 0.001.

We next compared currents in the presence and absence of the drug during a step depolarization protocol. Step currents were elicited using a series of 2-second depolarizations ranging from -50 mV to +60 mV (in 10 mV increments), and the subsequent tail currents were recorded during a 1.5-second repolarization step to -50 mV (Fig 2A). Typical bell-shaped current-voltage (I-V) curves for WT hERG were obtained when step currents were measured at the end of the depolarizing voltages (Fig 2B); the decline in current amplitude with depolarizing potentials was due to the fast inactivation [1,25]. As expected based on a previous study that demonstrated *R*-roscovitine’s preference to inhibit only open hERG channels [23], 200 μM *R*-roscovitine inhibition was weak at both lower voltages (due to low open probability) and at higher voltages (due to the strong inactivation). For example, at -50 mV inhibition at was only 23 ± 4.5% and at +50 mV it was only 8.2 ± 5.4%. In contrast, inhibition was significantly stronger at intermediate voltages (where inactivation was weak and open probability high), reaching a maximum of 43.3 ± 3.0% at 0 mV (Fig 2C).

Since hERG channels inactivate very rapidly during step depolarizations, hERG channel block is studied from tail currents [48,49]. Following the depolarization step, repolarization to -50 mV allowed hERG channels to be rapidly released from inactivation and transition to the open state, causing a large efflux of K^+^ ions (Fig 2A); these tail currents reflect the proportion of channels activated at the preceding depolarized potentials [25,50]. Plotting normalized peak tail currents against step voltages resulted in typical sigmoidal activation curves (Fig 2D), with a threshold for current activation of approximately -40 mV and a near-maximum channel activation at +40 mV. Activation curves were fitted with a Boltzmann equation, yielding a V_0.5_ of activation of -16.5 ± 1.1 mV, well in line with previous studies (e.g. [51]). Application of 200 μM *R*-roscovitine shifted the V_0.5_ of activation by a statistically significant ~ -3 mV (dashed line in Fig 2D). In addition, *R*-roscovitine inhibition of tail current significantly increased with depolarization from 30.9 ± 4.9% at -40 mV to 45.6 ± 3.1% at 0 mV, and reached its maximum of 52.3 ± 2.7% at +60 mV (Fig 2E). Thus, *R*-roscovitine inhibition of tail current increased with channel activation. This type of voltage dependence of inhibition is typical for drugs that bind to the open state, and is in line with previous studies [23].

### Comparison of WT and mutant hERG channels in the absence of *R*-roscovitine

**Fig 3:**
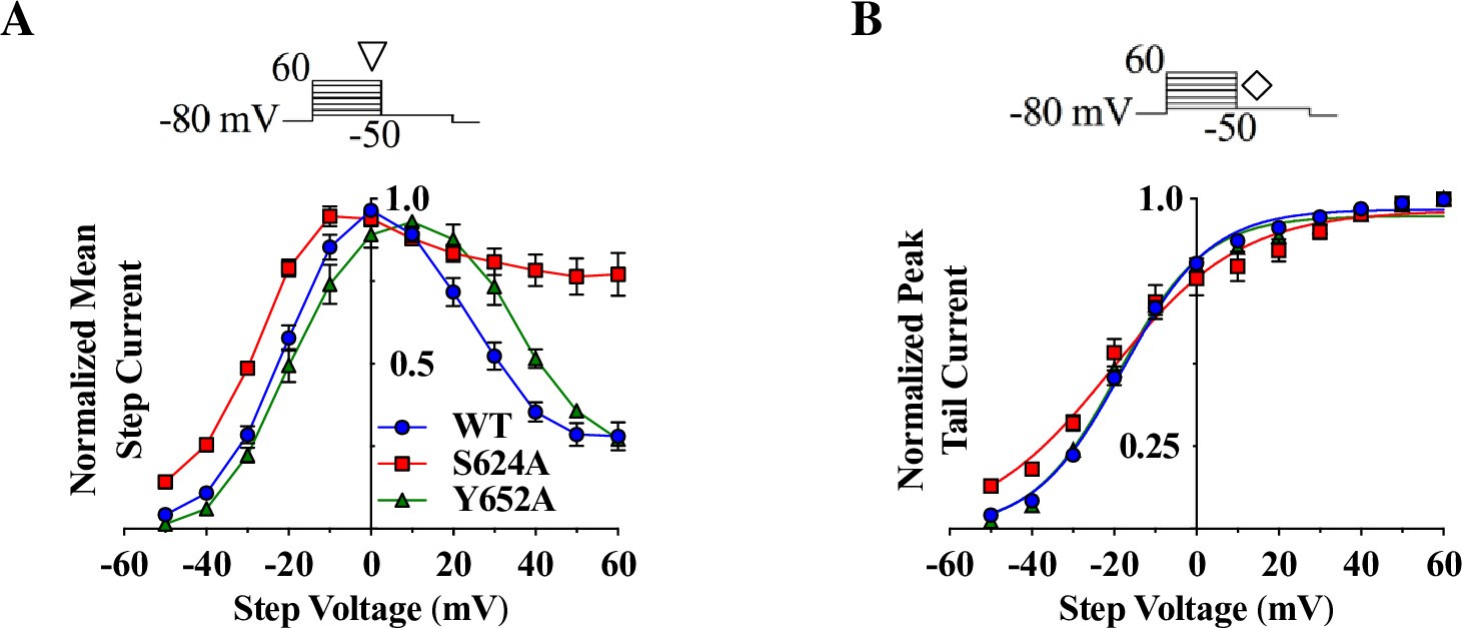
Comparison of unaffected step and tail currents from WT hERG, S624A, and Y652A hERG mutants. **A)** Step I-V curves for WT (○, blue), S624A (□, red), and Y652A (△, green) from a step depolarization protocol (top; currents measured at ▽). Rectification of S624A currents was reduced in comparison to WT current, with more than twice the amount of normalized current remaining for S624A (0.77 ± 0.06) compared to WT (0.28 ± 0.04) at +60 mV. **B)** WT, S624A, and Y652A tail I-V curves from a step depolarization protocol (top; currents measured at ◊). Boltzmann fits generated the following V_0.5_ of activation (neither of which were different from WT; p > 0.8 for WT-mutant comparisons): WT V_0.5_ = -16.5 ± 1.1 mV, S624A V_0.5_ = -17.7 ± 3.2 mV, and Y652A V_0.5_ = -16.5 ± 1.0 mV. The slopes for the activation curves were 11.7 ± 0.45 for WT, 16.3 ± 1.62 for S624A (p = 0.0033 compared to WT), and 12.5 ± 0.84 for Y652A (p = 0.7913 compared to WT); n_WT_ = 14, n_S624A_ = 9, n_Y652A_ = 9, one-way ANOVAs.

hERG block is commonly associated with residues located near the selectivity filter (e.g. S624) and on the S6 helices (e.g. Y652) [1,37,52]. Therefore, single-mutant channels S624A and Y652A were expressed in *Xenopus* oocytes and subjected to a step depolarization protocol (Fig 3A, top). Step I-V curves for WT, S624A, and Y652A showed maximum step currents at 0 mV, -10 mV, and +10 mV, respectively (Fig 3A, bottom). At +60 mV, S624A had more than twice the relative amount of current compared to WT channels, which indicated possible incomplete inactivation; this mutant channel was previously characterized to have a slower inactivation [53,54]. Boltzmann fits to the activation curves indicated no significant difference in half-maximal activation between S624A, Y652A, and WT (Fig 3B).

### The S624A mutation enhances, rather than inhibits *R*-roscovitine block

**Fig 4:**
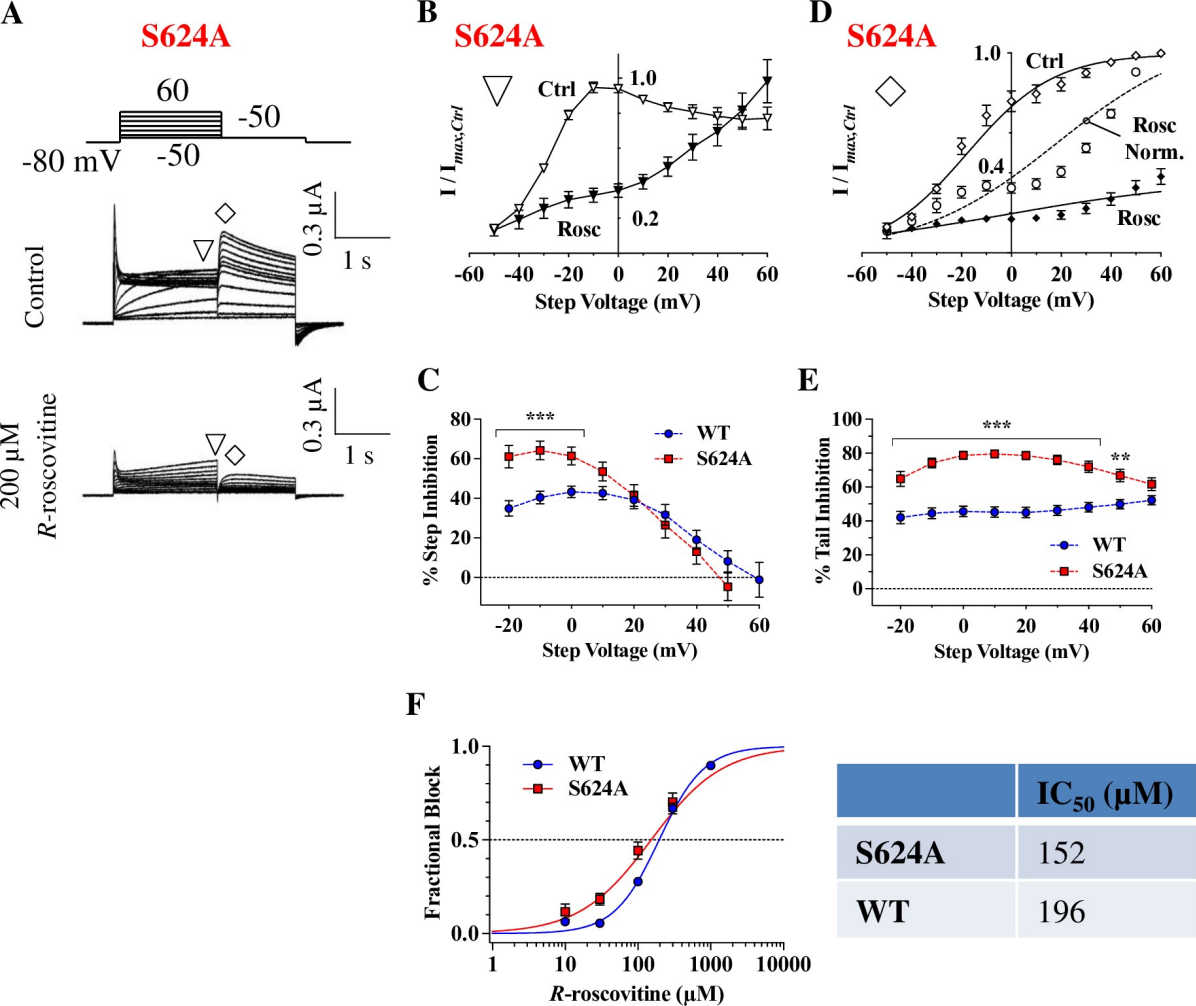
S624A hERG channels have an increased sensitivity to *R-*roscovitine. **A)** Voltage protocol and representative current traces, from a cell expressing S624A hERG, before and during 200 μM *R-*roscovitine application. Note that tail currents were greatly diminished upon *R-*roscovitine application. **B)** S624A step I-V curves before and during 200 μM *R-*roscovitine application (currents measured at ▽ in A). The strongest inhibition occurred between -30 mV and +30 mV. **C)** Percent step inhibition of WT and S624A at varying step voltages. S624A was inhibited more potently than WT at intermediate voltages, where open probability is high (p < 0.05 for WT vs. S624A between -20 mV and 0 mV, n_WT_ = 14, n_S624A_ = 9, one-way ANOVA). **D)** S624A tail I-V curves measured at ◊. Smooth lines are Boltzmann fits that generated V_0.5_ of activation in Control: -17.7 ± 3.2 mV and in *R-*roscovitine: -3.3 ± 6.1 mV; ns, p = 0.09. The slopes were 16.3 ± 1.6 in Control, and 44.5 ± 3.3 in *R-*roscovitine (p < 0.0001; n = 9, paired *t*-tests). Dashed line indicates the fit to normalized currents in *R-*roscovitine. **E)** Percent tail inhibition of WT and S624A. When compared to WT over a range of voltages, levels of S624A tail current inhibition were much larger (p < 0.05 for WT vs. S624A between -20 mV and +50 mV, n_WT_ = 14, n_S624A_ = 9, one-way ANOVA). **F)** Concentration-response relationship for S624A. The S624A IC_50_ (152 ± 22 μM) was not significantly lower than the WT IC_50_ (196 ± 12 μM;p = 0.8323 for WT vs. S624A) but the dose-response slope for S624A was statistically smaller (0.84 ± 0.08 vs 1.5 ± 0.1 for WT; p = 0.0003, n_WT_ = 11, n_S624A_ = 9, one-way ANOVA). ** = *P* < 0.01, and *** = *P* < 0.001.

In general, mutating key residues thought to be involved in drug-binding has been shown to weaken or abolish hERG drug inhibition [55]. To determine how changes to hERG pore residues alter *R*-roscovitine inhibition, WT and mutant channel currents were compared in the presence and absence of 200 μM *R*-roscovitine (Fig 4A). Applying the step depolarization protocol to S624A hERG channels resulted in currents with the step I-V curves shown in Fig 4B, and inhibition occurred at intermediate voltages (i.e. between -30 mV and +30 mV; Fig 4B). Surprisingly, mutating the S624 residue did not reduce 200 μM *R*-roscovitine current inhibition: S624A and WT step current inhibition were almost identical over a range of step voltages (p > 0.05 for WT vs. S624A between +10 mV and +60 mV; Fig 4C). In fact, at a few intermediate voltages (-20 mV and 0 mV), the inhibition of S624A was almost 25% larger than WT inhibition (p < 0.05, Fig 4C).

S624A tail I-V curves had typical sigmoidal curves and a V_0.5_ of activation of -17.7 ± 3.2 mV before *R*-roscovitine application (Fig 4D). Although there seemed to be a shift in activation V_0.5_ during *R*-roscovitine block of S624A, this shift was not statistically significant (p = 0.09; Fig 4D). The shift in the slope of the activation curve, however, was significant, indicating altered gating (Fig 4D). In addition, the very strong inhibition, combined with an apparent reduction in inhibition at depolarized voltages resulted in a poor sigmoidal fit (see discussion). Importantly, S624A tail current inhibition was significantly stronger than WT channel inhibition over a range of voltages (p < 0.05; Fig 4E). At +50 mV, S624A tail inhibition by 200 μM *R*-roscovitine reached 66.8 ± 3.6%, which was significantly larger than WT inhibition (49.9 ± 2.8%; p = 0.0012; Fig 4E). When we measured dose-response curves, S624A IC_50_ (152 ± 22 μM) was smaller than WT IC_50_ (196 ± 12 μM), but this difference did not reach statistical significance (Fig 4F). Nevertheless, the slope of the S624A IC_50_ curve was significantly smaller (0.8 ± 0.08 for S624A vs. 1.5 for WT; p < 0.001), perhaps indicating an allosteric change in the binding site, and reflecting stronger inhibition of S624A at some drug concentrations (Fig 4F). The persistence of inhibition suggested that S624 may not be required for *R*-roscovitine inhibition, and prompted further examination of other residues typically involved in hERG inhibition [56].

### The Y652A mutation weakens inhibition

**Fig 5:**
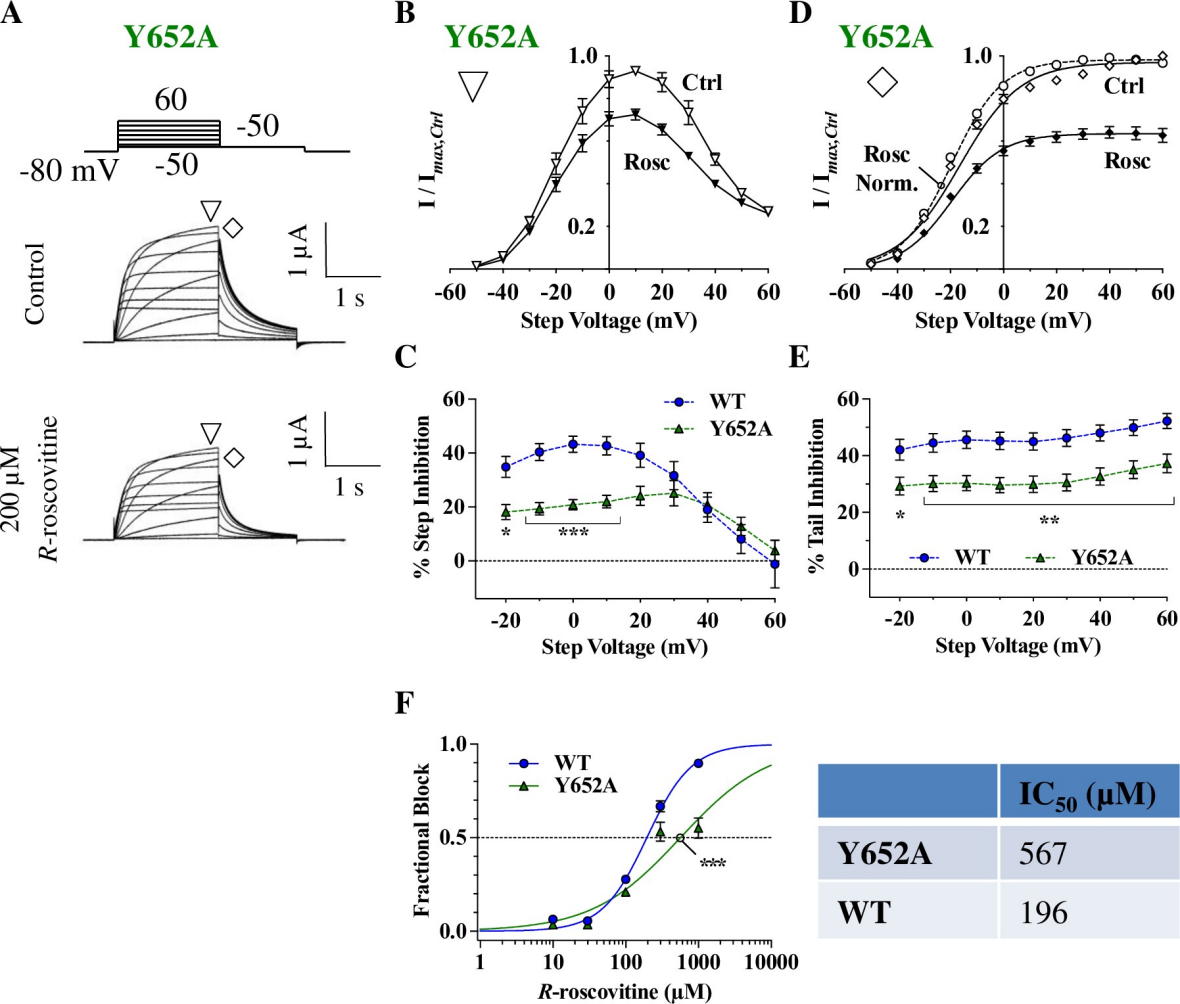
The Y652A mutation attenuates hERG inhibition by *R-*roscovitine. **A)** Voltage protocol and representative current traces of a cell expressing Y652A hERG before and during 200 μM *R-*roscovitine application. **B)** Y652A step I-V curves before and during 200 μM *R-*roscovitine inhibition (currents measured at ▽). The curves were bell-shaped and displayed inhibition at intermediate voltages. **C)** Percent step inhibition of WT currents were much stronger than that of Y652A between -20 mV and +10 mV (p < 0.05 for WT vs. Y652A, n_WT_ = 14, n_Y652A_ = 9, one-way ANOVA). **D)** Boltzmann fits of the Y652A tail I-V curves from the step depolarization protocol (currents measured at ◊) showed a significant shift in V_0.5_ and slopes for the Ctrl vs Rosc activation curves. Ctrl V_0.5_ = -16.5 ± 1.0 mV, and a Rosc V_0.5_ = -19.6 ± 0.5 mV (p = 0.0094), and activation slopes were 12.5 ± 0.8 for Ctrl and 9.3 ± 0.3 for Rosc. (p = 0.0025, n = 11, paired *t*-tests). Dashed line indicates the fit to normalized currents in *R-*roscovitine. **E)** Percent tail inhibition of WT and Y652A at their respective step voltages. Levels of Y652A tail current inhibition were significantly reduced compared to WT between -20 mV and +60 mV (p < 0.05 for WT vs. Y652A, n_WT_ = 14, n_Y652A_ = 9, one-way ANOVA). **F)** Concentration-response relationship for Y652A. When compared to WT IC_50_ (196 ± 12 μM), Y652A had a ~ 2.9-fold increase in IC_50_ (567 ± 122 μM; p = 0.0005). The slope of the dose response curve significantly changed from 1.522 ± 0.1 for WT to 1.0 ± 0.14 for Y652A (p = 0.0052; n_WT_ = 11, n_Y652A_ = 8, one-way ANOVAs). * = *P* < 0.05, ** = *P* < 0.01, and *** = *P* < 0.001.

We next tested the importance of the Y652 residue, which is a key binding target for several hERG inhibitors [57]. Using the same step depolarization protocol as before (Figs 2–4), step and tail currents were elicited in the presence or absence of 200 μM *R-*roscovitine (Fig 5A). Unlike S624A, Y652A hERG exhibited a statistically significant reduction in inhibition of step current compared to WT hERG (Fig 5B and 5C). Analogous to WT tail I-V curves, Y652A tail I-V curves were shifted ~3 mV to more hyperpolarized voltages by *R-*roscovitine (Fig 5D). Percent tail inhibition in Y652A was ~30% weaker than WT between -20 mV and +60 mV, and showed the highest levels of inhibition at large depolarized potentials (p < 0.05; Fig 5E). Furthermore, the Y652A IC_50_ (567 ± 122 μM) was ~ 2.9-fold larger than WT (196 ± 12 μM; p = 0.0005; Fig 5F). The attenuated step and tail current inhibition of Y652A with 200 μM *R*-roscovitine, as well as the difference in IC_50_ values, seemed to indicate that Y652 is involved in *R*-roscovitine inhibition. However, the relatively small reduction in IC_50_ with this mutation, compared to ~20 fold [34] or even larger reduction in IC_50_ [32] found with many other compounds [58], made us question whether additional residues in the pore region were involved in *R-*roscovitine inhibition, and whether they would be more critical.

### The high K^+^ solution alters *R*-roscovitine inhibition of WT hERG

In addition to the residues S624 and Y652, we wanted to test whether other pore residues are involved. In particular, T623, a residue adjacent to the selectivity filter, and F656, an S6 residue, both of which are commonly associated with hERG block [52]. Since these are pore residues, mutating them alters permeation such that inward rectification is significant, with very little outward currents present. Therefore, as previously established, a high K^+^ solution, coupled with inward tail current measurements at -120 mV, were required to investigate the T623A and F656A hERG mutants (see methods) [31,59–61].

**Fig 6:**
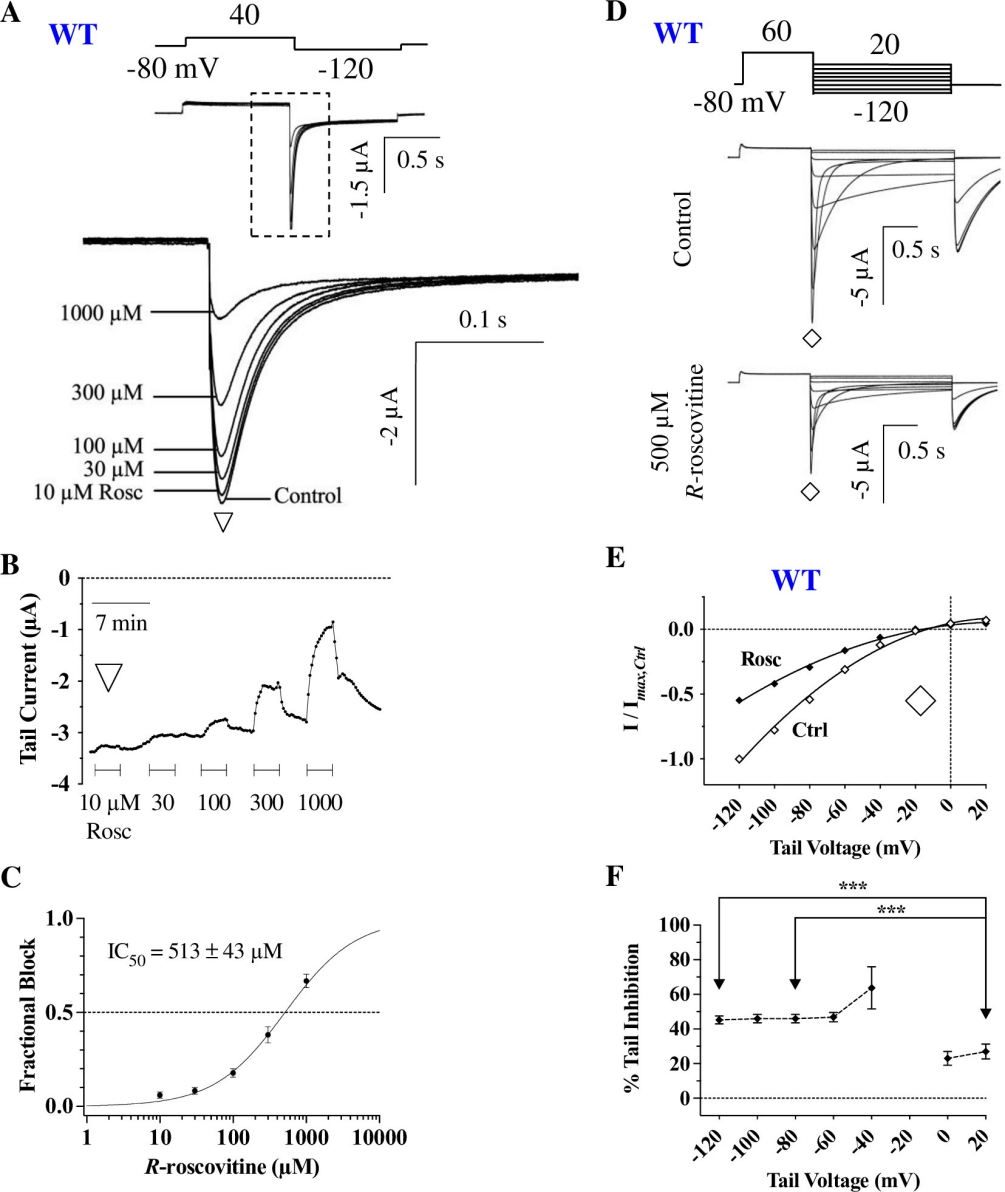
Weaker hERG current inhibition in the high K^+^ solution. **A)** Representative WT tail currents in high K^+^ prior to (Control) and during the indicated *R-*roscovitine concentrations, elicited by the protocol above. Tail currents from the dashed boxed region are expanded for clarity. **B)** Time course of inhibition from the cell in A. Peak tail currents were measured at ▽ during which the indicated *R-*roscovitine concentrations were applied and washed off. Peak tail currents were plotted over time. **C)** The IC_50_ for WT hERG in high K^+^ was 513 ± 43 μM (n = 9), which was significantly different from WT in low K^+^ (p <0.0001); the slopes (high K^+^ = 1.1 ± 0.2) were not (low K^+^ = 1.5 ± 0.1; p = 0.091, unpaired *t-*tests). **D)** Voltage protocol and representative current traces of a cell before (Ctrl) and during 500 μM *R-*roscovitine application. **E)** WT tail I-V curves from currents measured at ◊ in D. The smooth quadratic fits yielded a reversal potential (*E*_rev_) of -11.1 ± 3.3 mV in control, and -14.6 ± 2.4 mV in 500 μM *R*-roscovitine (p = 0.0237, n = 10, paired *t-*test). **F)** Percent tail inhibition calculated from E. Inhibition was significantly stronger at lower voltages than at high voltages (p = 0.0004 for +20 mV vs. -120 mV, p = 0.0002 for +20 mV vs. -80 mV, n = 10, one-way ANOVA). *** = *P* < 0.001.

Previous studies that utilized a high K^+^ solution to investigate hERG drug affinities have found that this condition increases the IC_50_. This is thought to occur due to the observed reduction in inactivation in high K^+^ (which would reduce the affinity of drugs that bind to the inactivated state) as well as due to a ‘knock-off’ effect, where drugs are pushed back into the cytoplasm by the inward K^+^ flux [30,34,60,62,63,63–65]. Thus, before studying T623A and F656A mutants, we sought to determine WT channel IC_50_ in the high K^+^ solution. A pulsing protocol was applied, comprised of a 1 second depolarization to +40 mV and a 1 second repolarization to -120 mV; peak tail currents were measured in various *R*-roscovitine concentrations (Fig 6A and 6B). As expected, the IC_50_ for WT channels was significantly higher in the high K^+^ solution (513 ± 43 μM; Fig 6C) compared to the low K^+^ solution (196 ± 12 μM). The 2.6 fold increase in IC_50_ was significant (p <0.0001), while the slope of the dose response curve was not (Fig 6C). 500 μM *R-*roscovitine was, therefore, used with a step repolarization protocol to compare WT tail currents prior to and during drug application (Fig 6D).

Measuring peak tail currents during repolarization showed that 500 μM *R-*roscovitine shifted the reversal potential from -11.1 ± 3.3 mV to -14.6 ± 2.4 mV (p = 0.0237; Fig 6E), indicating a slight change in hERG permeation properties with *R*-roscovitine block, as would be expected from compounds that bind to channels’ pores [66–68]. We also found a statistically significant, albeit smaller shift in reversal potential in the low K^+^ solution suggesting this is not a side-effect of the high K^+^ solution (S1 Fig). Furthermore, *R-*roscovitine inhibition increased as current direction changed from outward to inward: for example, WT hERG inhibition at both -120 mV (45.2 ± 2.3%) and -80 mV (45.9 ± 2.5%) was significantly larger than at +20 mV (27.0 ± 4.3%; p < 0.0005 for either comparison; Fig 6F). The shift in *E*_rev_ for both low K+ and high K^+^ experiments on WT hERG, along with the increased inhibition at hyperpolarized voltages, corroborated previous studies [23] suggesting that *R*-roscovitine binds to the open hERG pore.

### T623A and F656A mutations significantly disrupt inhibition

**Fig 7:**
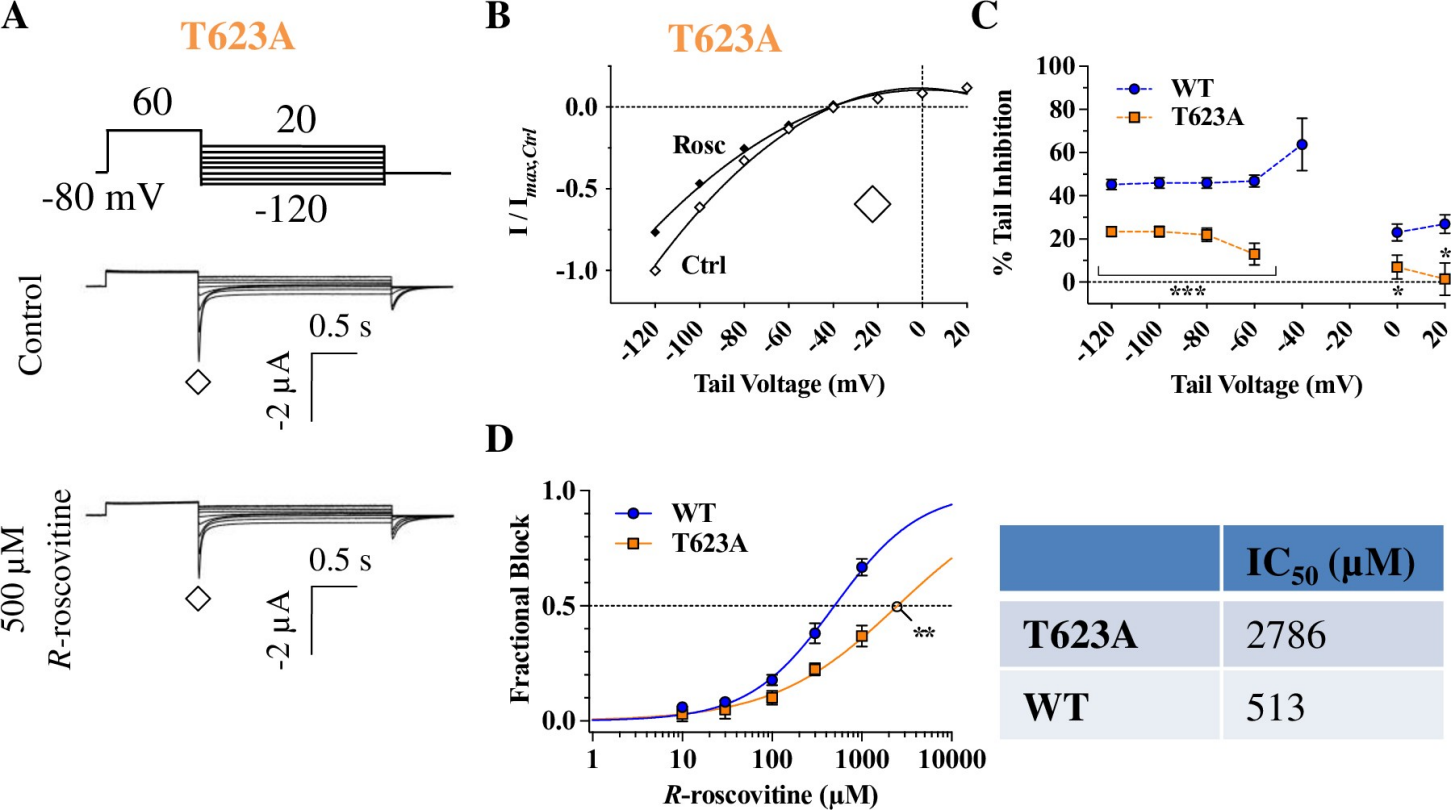
T623A hERG reduces *R-*roscovitine mediated inhibition. **A)** Voltage protocol and representative current traces of a cell expressing T623A before (Control) and during 500 μM *R-*roscovitine application. **B)** T623A tail I-V curves during the step repolarization protocol (currents measured at ◊). Quadratic fits generated *E*_rev_ values (Ctrl = -39.7 ± 2.0 mV, Rosc = -40.1 ± 2.1 mV) that did not show a significant shift occurring with 500 μM *R*-roscovitine (p = 0.5995 for Ctrl vs. Rosc, n = 10, paired *t-*test). **C)** Percent tail inhibition of T623A hERG was significantly weaker over a range of voltages than WT inhibition (p < 0.05 for WT vs. T623A between +20 mV and -120 mV (excluding voltages close to the reversal potential; n_WT_ = 10, n_T623A_ = 10, one-way ANOVA). **D)** Compared to the WT IC_50_ (513 ± 43 μM), T623A IC_50_ was ~ 5.5-fold larger (2786 ± 488 μM; p = 0.0092) while the slope factor had a non-significant change from 1.1± 0.22 for WT to 0.7 ± 0.09 for T623A (p = 0.4901; n_WT_ = 9, n_T623A_ = 7, Kruskal-Wallis tests). * = *P* < 0.05, ** = *P* < 0.01, and *** = *P* < 0.001.

*R-*roscovitine exhibited a very weak inhibition of T623A hERG tail currents during a step repolarization protocol (Fig 7A, 7B and 7C): between -60 mV and -120 mV, T623A inhibition was approximately half that of WT inhibition (p < 0.05; Fig 7C). Consistent with a significantly reduced inhibition by *R-*roscovitine, plotting T623A tail currents against tail voltages resulted in quadratic nonlinear fits that showed no shifts in *E*_rev_ in the presence of *R-*roscovitine (Fig 7B). Furthermore, the attenuated potency of *R-*roscovitine with T623A hERG was supported by IC_50_ value comparisons: T623A IC_50_ was ~ 5.5-fold higher than that for WT (p = 0.0092; Fig 7D). These results demonstrated the importance of T623 for *R-*roscovitine inhibition.

**Fig 8:**
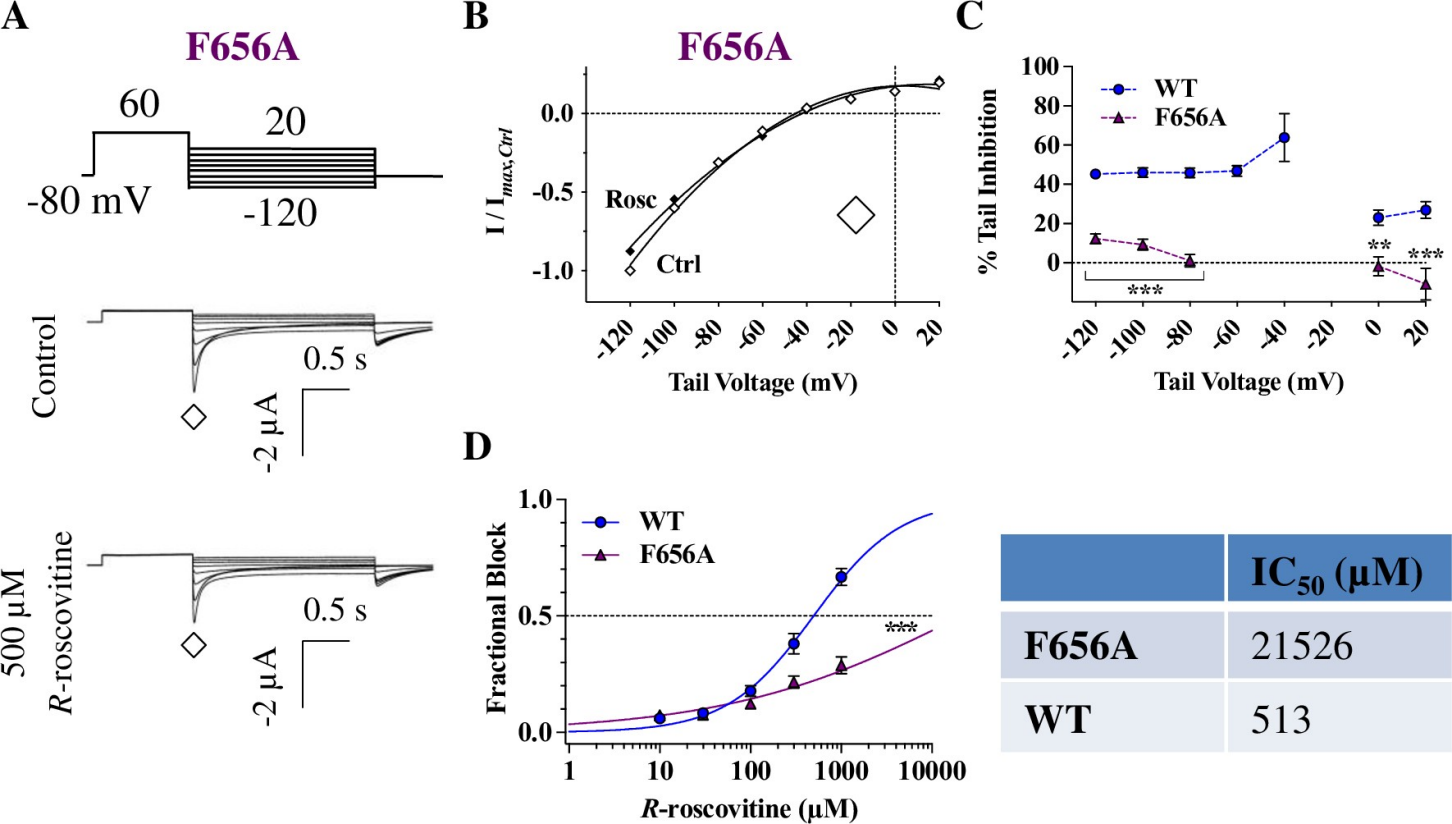
*R-*roscovitine inhibition is almost completely abolished in F656A hERG. **A)** Voltage protocol and representative current traces of a cell expressing F656A before (Control) and during 500 μM *R-*roscovitine application. **B)** F656A tail I-V curves during the step repolarization protocol (currents measured at ◊). Quadratic fits generated to obtain *E*_rev_ values (Ctrl = -45.1 ± 1.1 mV, Rosc = -42.1 ± 1.7 mV) did in fact show a shift with drug block (p = 0.02, n = 9, paired *t-*test). **C)** F656A tail current inhibition was almost non-existent for most repolarized voltages, which was a substantial difference from WT inhibition (p < 0.01 for WT vs. F656A between +20 mV and -120 mV (excluding points close to the reversal potential), n_WT_ = 10, n_F656A_ = 9, one-way ANOVA). **D)** Concentration-response relationship for F656A hERG IC_50_ (21.5 ± 10.6 mM) shows a ~ 42-fold increase from WT IC_50_ (513 ± 43 μM; p = 0.0004); and a significant reduction in the slope of the Hill equation from 1.1 ± 0.22 for WT to 0.42 ± 0.04 for F656; p < 0.005; n_WT_ = 9, n_F656A_ = 5, Kruskal-Wallis tests. ** = *P* < 0.01, and *** = *P* < 0.001.

The F656A mutation (located in the S6 helix) exhibited the largest level of resistance to 500 μM *R-*roscovitine and tail currents were minimally impacted (Fig 8A). This initial observation extended to most tail voltages during repolarization (Fig 8B). At -120 mV, *R-*roscovitine inhibition of F656A hERG was only 12.3 ± 2.5%, compared to the 45.2 ± 2.3% inhibition of WT hERG (p < 0.001; Fig 8C). Surprisingly, there was a small but statistically significant shift in F656A *E*_rev_ by 500 μM *R-*roscovitine (from -45.1 ± 1.1 mV to -42.1 ± 1.7 mV for Ctrl vs. Roscovitine, respectively; p = 0.02). The IC_50_ for F656A was increased ~ 42-fold from WT (Fig 8D), and the slope of the IC_50_ curve was dramatically reduced (Fig 8D), consistent with the incomplete inhibition [69]. These results, as well as the results for T623A hERG (Fig 7), indicated drastic modifications to the *R-*roscovitine binding site with mutations to either one of these hERG pore residues.

**Fig 9:**
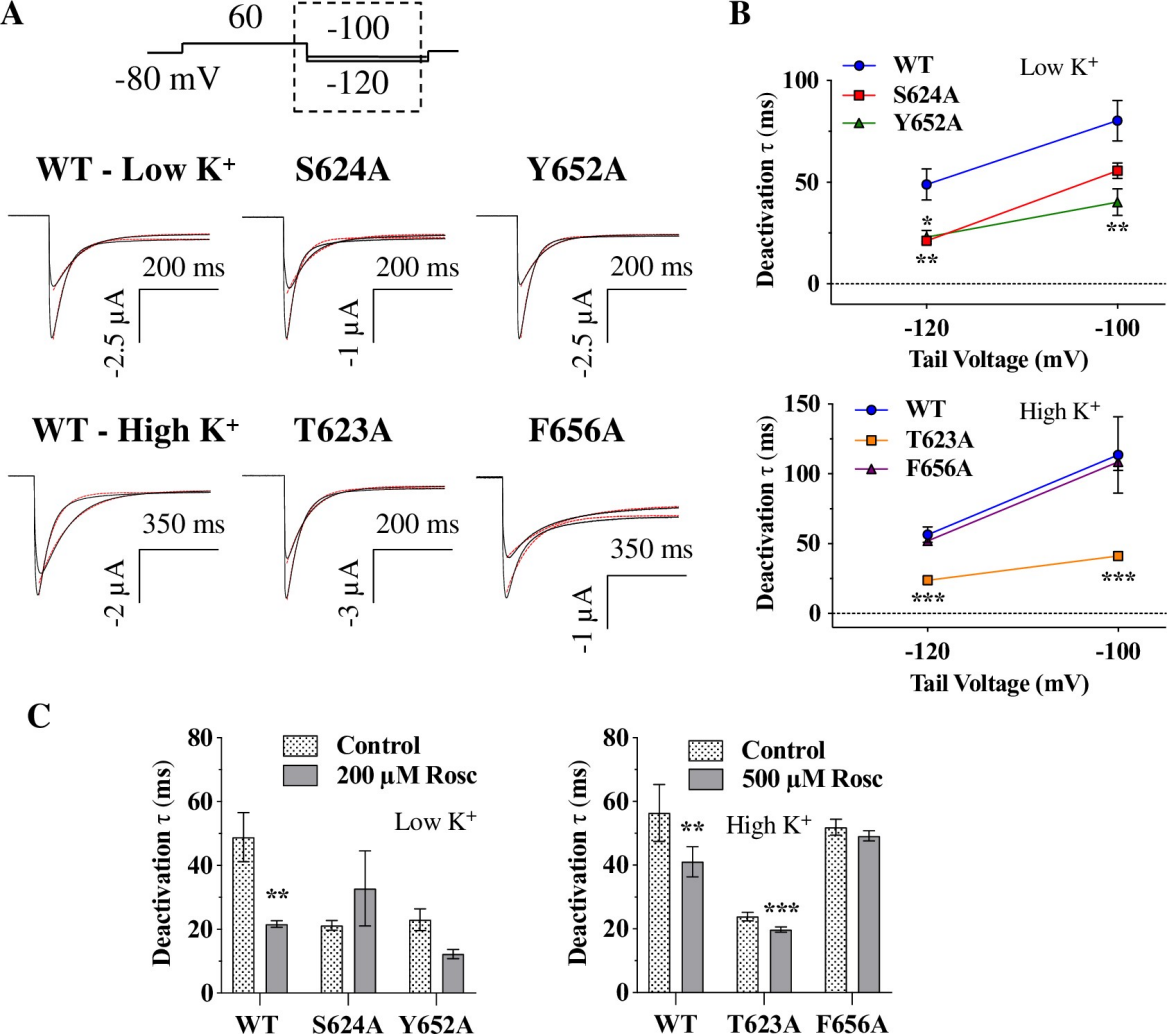
Deactivation time constants for hERG and its mutants. **A)** Representative traces from the indicated hERG constucts at -100 and -120 mV repolarization voltages. Tail currents (from the dash-boxed region of the voltage protocol) were fitted with a standard biexponential equation (red dashed line) to measure deactivation time constants (see methods). **B)** Average τ_fast_ for S624A, Y652A and T623A constructs at -120 mV were all significantly smaller than their respective WT controls (p = 0.0082, p = 0.0134, and p < 0.0001 respectively). At -100 mV, Y652A and T623A were significantly different from their respective WT controls (p = 0.0054 and p = 0.0009), while S624A was not (p = 0.0953). F656A was not different from WT_High K+_ (p = 0.4973 at -120 mV and p = 0.9430 at -100 mV). N = 6–10; one-way ANOVAs. **C)** τ_fast_ before (Control) and during *R-*roscovitine application at -120 mV. *R-*roscovitine sped up deactivation for WT (p = 0.0034) and T623A (p = 0.0009), but not for S624A (p = 0.312), Y652A (p = 0.2739), or F656A (p = 0.1708). N = 6–10; paired *t*-tests. *R-*roscovitine concentration was 200 μM for low K^+^ and 500 μM for high K^+^ solutions. * = *P* < 0.05, ** = *P* < 0.01, and *** = *P* < 0.001.

Since *R-*roscovitine binds to the open state, any mutations that change gating could potentially alter inhibition allosterically, rather than disrupt binding with *R-*roscovitine. Therefore, mutations that prolong channel opening may be expected to enhance inhibition, while those that reduce channel opening could be expected to decrease channel inhibition. Fig 9 shows deactivation time constants for WT and mutant hERG channels. As initially suspected based on the tail currents in Fig 5A, Y652A deactivated significantly faster than WT channels (Fig 9A and 9B). Thus, we cannot rule out that this gating change is at least partly responsible for the reduced inhibition by *R-*roscovitine. In contrast, we find that the S624A mutant deactivates either the same (at -100 mV) or faster than WT channels (at -120 mV; Fig 9), yet this mutant is inhibited more potently than WT channels (Fig 4). The T623A mutant had a significantly faster deactivation than WT, and the F656A mutant had deactivation kinetics similar to WT, yet they both exhibited dramatically reduced inhibition by *R-*roscovitine (Figs 7 and 8). This, coupled with other hERG mutant properties suggests that *R-*roscovitine likely binds directly to the investigated pore residues, as has been suggested for almost all other hERG inhibitors ([56]; see discussion).

Drugs that bind to the closed or inactivated state of hERG usually slow deactivation by a ‘foot in the door’ mechanism [70,71]. Since *R-*roscovitine would not be expected to slow deactivation, we examined its effect on deactivation time constants. As Fig 9C shows, WT deactivation is indeed faster, rather than slower, in the presence of *R-*roscovitine. Interestingly, T623A also showed a statistically significant speeding of deactivation in the presence of *R-*roscovitine, while the other hERG mutants exhibited no change in deactivation.

### *R*-roscovitine docking identifies residues likely involved in hERG inhibition

**Fig 10:**
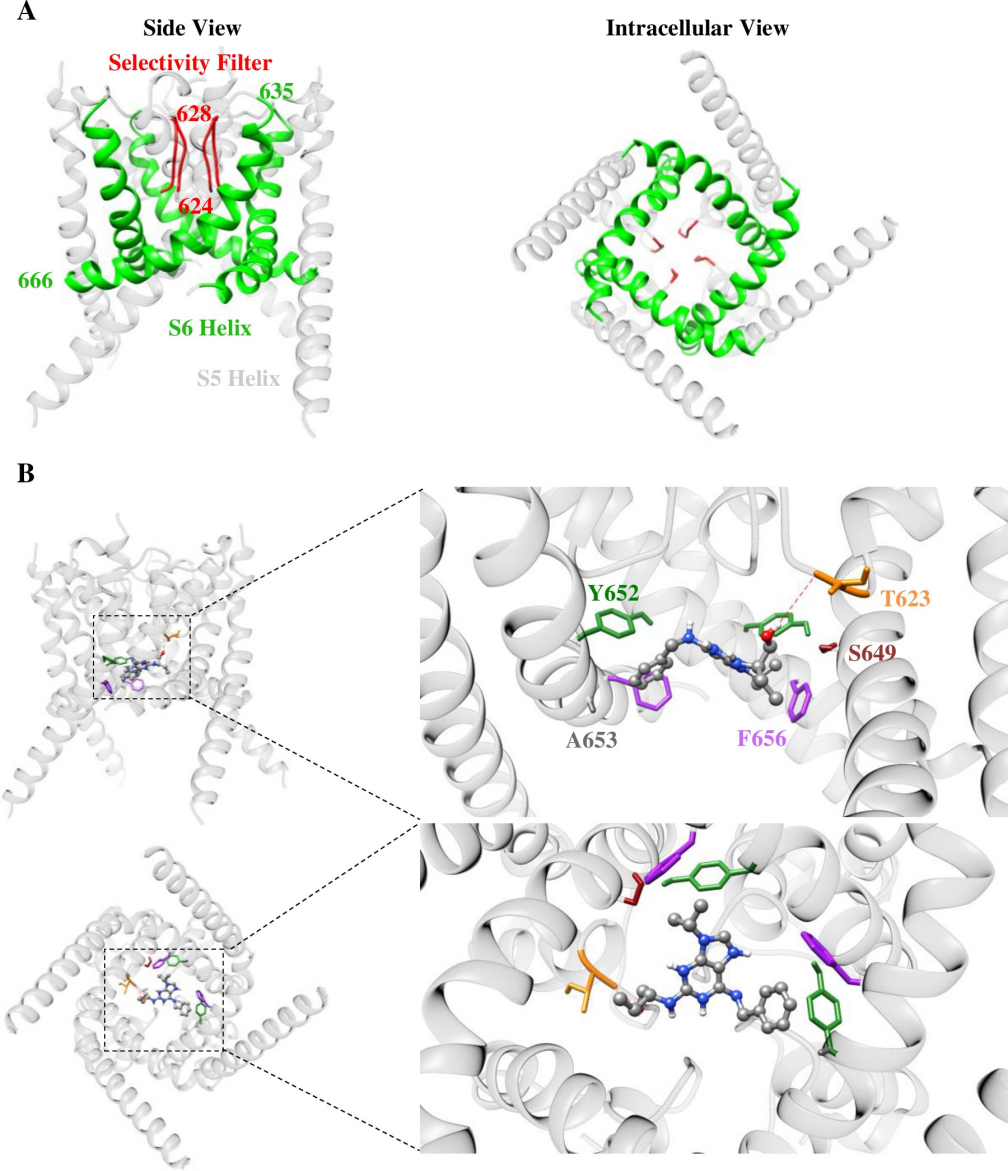
Molecular docking predicts *R*-roscovitine binding to the hERG selectivity filter and S6 helix residues. **A)** Structure of an open WT hERG homology model [43] based on the KvAP structure [44], with the selectivity filter and S6 helices highlighted in red and green, respectively (left = side view, right = intracellular view). 624–628 are selectivity filter residues and 635–666 are S6 residues. **B)** Binding site of a lowest-energy *R*-roscovitine conformation (ball & stick) shown in side-view and an inside-out view through the channel (see docking methods). Colored residues (stick) interacted with *R*-roscovitine using van der Waals interactions. Hydrogen bonding (red dashed-line) occurred between the backbone carbonyl oxygen of T623 and *R-*roscovitine. hERG subunit D, which did not interact with *R-*roscovitine, was removed for clarity in the zoomed-in side view (top panel). The residue color scheme is the same in both views.

Next, in order to provide an independent line of investigation into hERG residues likely involved in *R-*roscovitine-mediated inhibition, we conducted docking studies. To identify the *R-*roscovitine binding site and key hERG residues involved, *R-*roscovitine was docked to a hERG homology model [37] (Fig 10A; see methods) based on the KvAP crystal structure [37,38,72], and using Autodock Vina [73,74]. Fig 10B shows a lowest energy conformation where *R*-roscovitine is docked to the hERG pore, and interacts with residues T623, Y652, F656, but not S624. This conformation corroborates our experimental observations. Two Y652, and two F656 residues appear to bind to *R-*roscovitine, likely explaining their significant role in inhibition (Fig 10B). T623 appears to use hydrogen bonding to interact with *R-*roscovitine (Fig 10B), in accord with the relatively strong observed reduction in T623A inhibition (Fig 7). In our docking simulation, *R-*roscovitine was capable of taking five additional lowest energy conformations within the pore that were less consistent with our experimental observations (see discussion and S1 Table). In all conformations, however, *R-*roscovitine appears to bind in the inner central cavity, reaching both S6 residues (Y652 and F656) and residues near/in the selectivity filter (T623 and S624). In addition to the residues we tested experimentally, residues S649 and A653 appeared to bind *R-*roscovitine; they appeared as binding partners in all conformations (Fig 10B; S1 Table).

A recent cyro-EM hERG structure became available [10]. This structure was used for docking with various hERG blockers and was successful in reproducing experimental data after minor adjustments to the structure [61]. We docked *R-*roscovitine into the unmodified cryo-EM structure but obtained results that were not supported by our experimental observations (see S3 Fig). Specifically, R-roscovitine could not reach T623, a residue likely implicated in *R-*roscovitine binding (Fig 7). Therefore, we did not pursue further analyses with this structure.

## Discussion

In this study we investigated the mechanisms of *R-*roscovitine-mediated inhibition of hERG channels in *Xenopus* oocytes and made several contributions: 1) we determined that the IC_50_ for *R-*roscovitine is ~200 μM (in low K^+^), 2) mutation of the F656 residue nearly abolishes inhibition, and the T623 residue seems critical for inhibition, 3) the Y652A mutation moderately reduces inhibition, and 4) S624 is not required for inhibition, and instead, S624A unexpectedly enhances inhibition. Docking analyses further supports our experimental data. These results, collectively, suggest a comparatively unique binding orientation for *R*-roscovitine within hERG’s pore.

First, the IC_50_ we obtained for *R-*roscovitine in *Xenopus* oocytes (~200 μM, Fig 1D) is ~ 7 fold higher than that found in HEK cells (27 μM; [23]). This, is expected because oocytes have a vitelline membrane barrier, and a yolk that acts as a sink for drugs [45]. In fact, hERG studies that have compared frog oocyte versus mammalian cell IC_50_ values typically find a 10–30 fold higher IC_50_ in *Xenopus* oocytes [45–47]. Importantly, the micromollar IC_50_ is within the range of *R-*roscovitine concentrations that are clinically achievable in blood plasma, and that have been clinically tested; these concentrations were also shown to reduce growth, or induce apoptosis of multiple myeloma, lung carcinoma, and other cell lines [18,19,75–80].

Competition between hERG drugs and K^+^ ions for electrostatic interactions within the pore have recently been suggested to contribute to the reduced affinities drugs have in high K^+^ solutions [59]. It is possible that this is the reason we observe a reduced affinity of *R*-roscovitine in hERG in the high K^+^ solution (Fig 6). Indeed, reduced affinities in the presence of a high K^+^ solution have been found both for drugs that are trapped in the pore [81], and those that are not (e.g. cisapride [82] and quinidine [65]). The drugs in the latter group experience a ‘knock-off’ effect by the large inward currents, reducing apparent drug affinity for hERG. Drugs in the former group may experience both a ‘knock-off’ effect and a loss of binding due to the reduced inactivation in high K^+^ solutions [30,34,60,62,63,63–65].

Second, *R*-roscovitine was expected to have a unique mechanism of inhibition when compared to other hERG inhibitors because of its preferential binding to open hERG channels and the properties outlined above [23]. Our data suggests this is likely the case since F656 and T623 are critical components of *R-*roscovitine inhibition, while S624 seems dispensable, and Y652 contributes a seemingly weak role in *R-*roscovitine mediated inhibition. The weak contribution of residue S624, together with the inability of *R-*roscovitine to be trapped in the pore [23], likely contribute to its lower affinity to hERG, as has been suggested in a study on vesnarinone [47]. Furthermore, the indispensability of S624, but not T623 is unlike the vast majority of other drugs for which the importance of these residues had been investigated. For example, MK-499 mediated hERG inhibition is weakened by a mutation to either S624A or Y652A. A weakening of block with either S624, Y652, or F656 mutations were observed for many drugs, such as nifekalant [83], terfenadine, cisapride, [82], clofilium, ibutilide [84], bupivacaine [54], and propafenone [85], to name a few. This makes *R-*roscovitine a relatively unique hERG inhibitor, and together with its demonstrated effectiveness against several types of cancers, worthy of further exploration.

### The role of specific pore residues in hERG-*R*-roscovitine inhibition

#### Y652 and F656

The first S6 mutant to be characterized with *R-*roscovitine was Y652A hERG. 200 μM *R-*roscovitine inhibited both step and tail currents (Fig 5A and 5B), as well as caused a negative shift in activation curves (Fig 5D). This shift in the presence of *R*-roscovitine, which is similar for both WT and Y652A, implicates *R-*roscovitine in open state stabilization/binding [86]. Interestingly, the Y652A mutation caused a modest ~2.75-fold increase in IC_50_ value (from ~200 μM to ~ 570 μM; Fig 5), suggesting that other residues may be more critical for *R-*roscovitine binding. In addition, since we (Fig 9B) and others [63] have observed a faster deactivation in this mutant, it is difficult to rule out the possibility the Y652A mutation contributes to reducing channel inhibition allosterically, even if Y652A activation was not altered (Fig 5D). On the other hand, an overwhelming majority of studies identify Y652 as critical for binding hERG inhibitors [26,31,57], and our modeling shows that two Y652 residues interact with R-roscovitine (Fig 10), which would predict a larger shift in IC_50_ in the Y652A mutant. Perhaps reconciling these differences, the Y652A mutant is proposed to have a re-oriented F656 residue that can bind and stabilize some hERG inhibitors [58]. This is thought to occur for drugs that, like *R-*roscovitine, have only a slight increase in IC_50_ in the Y652A mutant (e.g. bepridil, thioridazine, and propafenone [58]). In contrast, drugs that have a 40–650 fold increased IC_50_ in the presence of the Y652A mutation (such as cisapride, dofetilide, E-4031, MK-499 and many others) are thought to be either too small or too inflexible to interact with the re-oriented F656 [58]. This rescue of drug binding in the Y652A mutant [by the F656 residue] could explain both the small change in IC_50_ in the Y652A mutant, as well as the prominence of the Y652 in the docking simulations (Fig 10; S1 Table). While this scenario would be interesting to explore in simulations with *R-*roscovitine and Y652A hERG, the results and analyses above suggested that F656 could be far more critical for inhibition.

Indeed, the F656A hERG S6 mutant, displayed a near complete insensitivity to *R-*roscovitine and ~42 fold reduced IC_50_ (Fig 8). This is unlikely to be caused by allosteric effects since the change in F656A gating (a negative shift in activation [31] and slowed deactivation [63]) are expected to enhance rather than abolish inhibition. (To a similar effect, deactivation was not significantly different from WT in oocytes; Fig 9). This is in line with findings from numerous other studies implicating F656 as a hERG drug binding determinant [9,24,25,56]. These results, together with our modeling (Fig 10, and S1 Table) identified F656 as a critical residue for *R-*roscovitine binding.

A previous study on mexiletine, a type Ib antiarrhythmic agent, had also found a stronger effect of the F656A compared to the Y652A hERG mutation to reduce inhibition (~28-fold versus ~12 fold reduction in IC_50_ in relation to WT, respectively [87]). Furthermore, like *R-*roscovitine, mexiletine seems to preferentially bind to the open state, with little or no preference for the closed or inactivated state. However, mexiletine slows deactivation while *R-*roscovitine significantly speeds it (Fig 9C). It is possible that *R-*roscovitine binding to the pore alters voltage sensor movement, especially since the hERG voltage sensor (S4) is known to be functionally linked to the pore [10,88]. Indeed, our results suggest that hERG pore residues S624, Y652, and F656 seem required for the speeding of channel deactivation by *R-*roscovitine (Fig 9C). Thus, it would be intriguing to obtain a better pharmacophoric comparison between *R-*roscovitine and mexlietine, which could shed light on the coupling between the pore and S4.

#### T623 and S624

Residues T623 and S624 are positioned deep within the hERG pore, specifically at the base of the pore helix [25] (Fig 10). With both side chains containing hydroxyl groups, hydrogen-bonding is frequently implicated in their interactions with drugs [31,84,89]. Depending on the orientation and structure of drugs that bind to the pore, mutating these pore residues can impact intermolecular interactions. This was evident with the T623A hERG mutant, which exhibited an overall weak tail current inhibition by *R-*roscovitine, and a significantly larger IC_50_ (Fig 7). This indicates the drug was no longer able to bind in its original location, perhaps due to the requirement of hydrogen-bonding to T623 for maintaining normal potency. Alternatively, T623A may allosterically reduce *R-*roscovitine potency, since we observed intrinsically faster deactivation in the T623A mutant; this would reduce open time and *R-*roscovitine binding. In fact, allosteric effects of the T623A mutation have been recently suggested by studies that found attenuated inhibition in T623A mutants but no simultaneous binding to F656/Y652 *and* T623 residues in docking studies, suggesting that T623 may be inaccessible from the pore [37,90]. In contrast, many studies have suggested that T623 binds directly to hERG inhibitors. This includes studies on nifekalant [83], clofilium and ibutilide [84], ranolazine [59], amiodarone [34], and many other drugs [91]. In accord with these studies, our docking suggests that *R-*roscovitine hydrogen bonds with T623 (as well as binds to F656 and Y652 simultaneously; Fig 10, S1 Table).

Surprisingly, step and tail current inhibition were not reduced in the S624A mutant (Fig 4B and 4D). In fact, inhibition was stronger when compared to WT inhibition at some voltages (Fig 4C and 4E). These results suggest that S624 is not a likely binding determinant for *R-*roscovitine. Aside from the mutagenesis results, this is supported by the lowest energy conformation shown in Fig 10. Other alternatives exist, however, for a role of S624: First, a possible interaction with S624 may be too weak for the S624A mutation to disrupt binding; indeed, some other low energy docking conformations we obtained showed S624-*R-*roscovitine interactions (S1 Table). Second, the recently proposed reorientation of the S6 helix in the S624A mutant [61], may allosterically enhance *R-*roscovitine’s binding to the pore. Third, drug unbinding could be reduced in S624A, since this residue has been previously implicated in the dissociation of hERG blockers (e.g., recovery from propafenone-mediated inhibition of hERG was significantly reduced in the S624A mutant [85]). Finally, altered inactivation in the S624A mutant [53,54], may have enhanced inhibition. But this is unlikely to occur since a non-inactivating S620T mutant does not exhibit stronger inhibition [23], and considering that inactivation is removed from tail currents. While these alternatives remain to be explored, to the best of our knowledge, *R-*roscovitine appears to be unique in exerting a much stronger inhibition on S624A than WT channels: tail current inhibition was nearly doubled for some voltages (Fig 4E). This, in addition to the effects on activation (Fig 4) likely contributed to the unusual shape of the IV curve in the presence of *R-*roscovitine. Interestingly, we did not detect a statistically significant decrease in IC_50_, but the slope of the dose response curve was significantly reduced, reflecting the fact that smaller *R-*roscovitine concentrations exerted stronger inhibition (Fig 4). Another aspect of *R-*roscovitine’s inhibition of S624A is that there appeared to be some reduction in *R-*oscovitine block at higher voltages, which was visible in both step and tail IV curves (Fig 4). This, along with the dependence of block on the direction of current (see S1C Fig) are consistent with pore block, but future studies are warranted to explore the mechanisms by which *R-*roscovitine binds to the S624A pore to modulate the already aberrant S624A gating (see Figs 3 and 4; [53,54]).

In summary, our experimental results suggest that *R-*roscovitine likely has a unique binding orientation within the open WT hERG channel, relying on S6 residues (Y652, F656) and T623 but not S624 (a selectivity filter residue). This is, indeed, unlike many other hERG inhibitors mentioned above and may motivate future structural and *in silico* studies.

### Physiological relevance

Known hERG inhibitors linked to drug-induced LQTS, such as terfenadine, cisapride, astemizole, and sertindole, have been withdrawn from the market due to their inhibition of hERG channels and the associated side-effects [92]. The initial intrigue of *R*-roscovitine for this study was that a clinical trial using a range of *R-*roscovitine concentrations did not find QT prolongation or other cardiac side effects [18], such as those commonly seen with other hERG inhibitors. Thus, studies on *R-*roscovitine-hERG interactions may shed light on desirable drug features that could be used to identify potentially life-saving drugs that were never tested clinically due to hERG block.

Likely factors that contribute to the lack of arrhythmogenic side effects with *R-*roscovitine are its potency, and its pleiotropic effects. First, *R*-roscovitine has a low affinity for hERG that may limit its adverse effects, even though the values used in this study and in other research are close to clinically relevant doses (10–30 μM) [28]. Indeed, clinical trials for Cushing’s disease and malignant solid tumors used effective *R-*roscovitine concentrations of ~10 μM [18,19]. This is close to *R-*roscovitine’s IC_50_ in HEK cells (27 μM; [23]). Furthermore, *R-*roscovitine specifically targets proliferating versus non-proliferating cells in an array of 19 cancer cell lines, with an average IC_50_ of ~16 μM. It also induces significant tumor suppression in mice [75–77], as well as significant growth delay of human xenografts in mice [78]. Finally, it reduces growth of lung cancer, neuroblastoma cell lines, and two colon cancer cell lines (in the 20–100 μM range, [79,80]). Thus, when used either experimentally or clinically, *R-*roscovitine is expected to inhibit hERG channels. In comparison with other hERG inhibitors, however, the relatively low affinity of *R*-roscovitine likely stems from its selective binding to the open state [23], as high affinity hERG inhibitors tend to bind to the inactivated hERG state [93]. Indeed, *R-*roscovitine inhibition of the inactivation-deficient mutant S620T was not different from WT inhibition, suggesting low or no affinity for the inactivated state [23]. Furthermore, this drug is unique when compared to class III antiarrhythmic drugs that, unlike *R*-roscovitine, exhibit: 1) binding to selectivity filter residues [16,31,94], 2) have relatively slow kinetics of recovery from inhibition, 3) are trapped in the hERG pore, and 4) exhibit use-dependence [23,81,95,96] and/or reverse-use dependence [65]. Our results provide a molecular basis for the observed unique pharmacological properties of *R-*roscovitine and provide impetus for further studies.

Another key factor that may explain the lack of arrhythmogenic side effects could be the ‘multiple ion channel block effect’ [97], whereby the effect of hERG inhibition to prolong cardiac action potentials is offset by a compensatory action on another ion channel [23]. Indeed, *R*-roscovitine inhibits L-type calcium channels with a potency in the ~25 μM range [20,98], which would shorten cardiac action potentials, and oppose the excitatory effect of hERG blockade [23,99]. As a result, the re-classification of *R-*roscovitine as a Class VI antiarrhythmic agent was recently proposed due to its selective non-peak block of the late I_Ca,L_current, which likely averts cardiac arrhythmias [100,101]. Thus, revisiting drugs that were deemed clinically unsafe due to hERG block, but exhibit compensatory drug actions, may yield new pharmaceutical therapeutics.

## Supporting information

S1 Fig Step repolarization protocols for WT, S624A, and Y652A in the presence or absence of R-roscovitine. (PDF)

S2 Fig Conformations #1 and #4 of R-roscovitine docked into an open hERG channel. (PDF)

S3 Fig R-roscovitine docking in the cryo-EM structure. (PDF)

S1 Table Six lowest energy conformations identified from *R*-roscovitine docking. (PDF)
