# Supplementary material for: The molecular determinants of R-roscovitine block of hERG channels
Source: PLoS One. 2019 Sep 3;14(9):e0217733. doi: 10.1371/journal.pone.0217733 (PMC6719874; doi:10.1371/journal.pone.0217733)
Supplement: S1 Fig — (PDF) [file pone.0217733.s001.pdf]

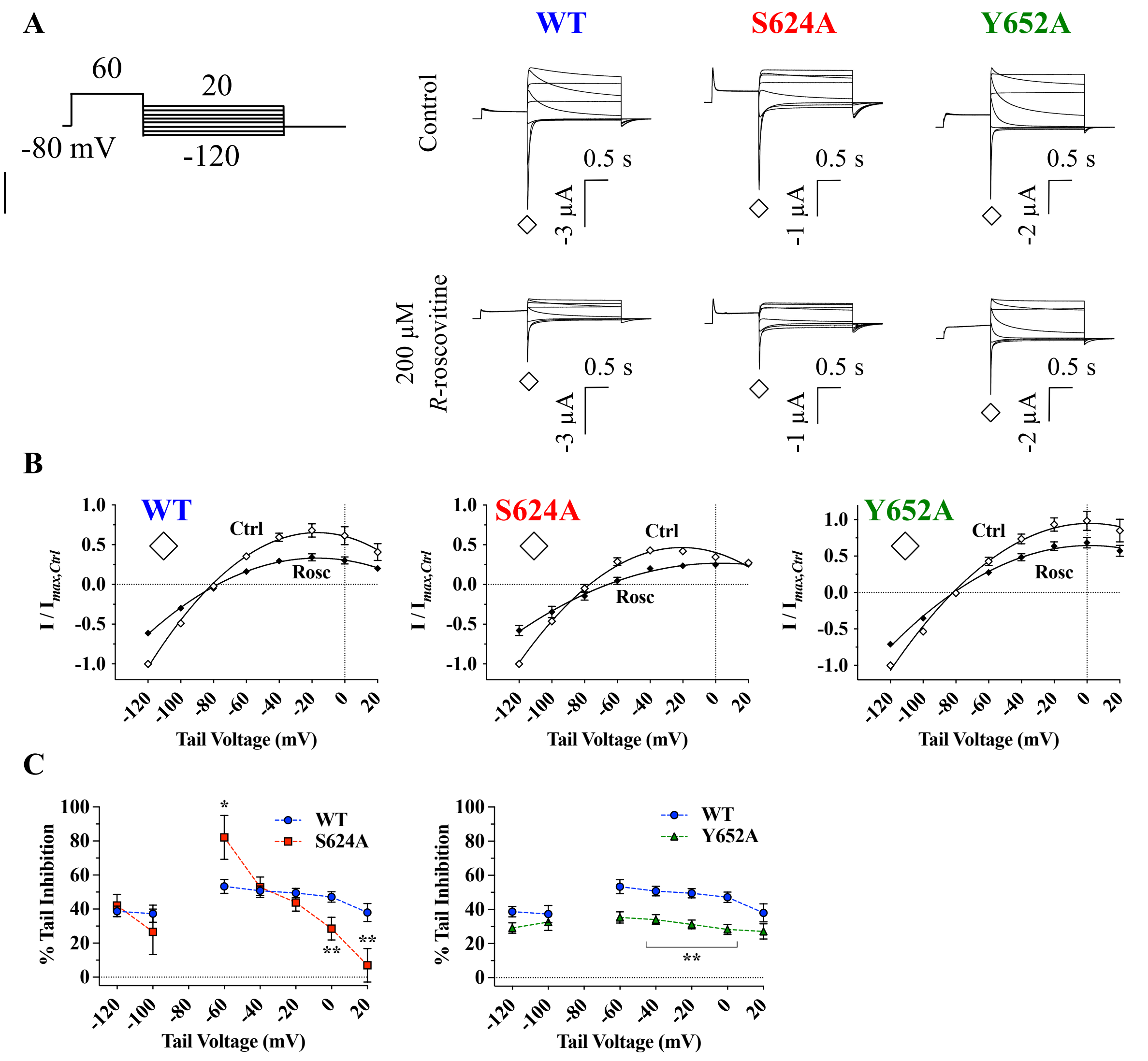

Supplementary Figure 1. Step Repolarization Protocols for WT, S624A, and Y652A in the presence or absence of *R*-roscovitine.

A) Representative current traces elicited from the step repolarization voltage protocol shown to the left. WT, S624A, and Y652A tail currents were measured at their peak (◇) in the absence (top row) and presence of 200 µM *R*-roscovitine (bottom row). **B)** Maximal tail current amplitudes from A were plotted against the repolarization voltage to show reversal potentials, which were then compared between control and *R*-roscovitine. The shifts for WT (E_{rev} = -80.16 ± 1.31 to -76.7 ± 1.33 mV; p = 0.0294, paired *t*-test) and S624A (E_{rev} = -82.25 ± 1.27 mV to -77.17 ± 2.00 mV; p = 0.0301, paired *t*-test) were significant, but not for Y652A (E_{rev} = -81.08 ± 1.28 mV to -80.61 ± 2.12 mV; p = 0.6238, paired *t*-test). **C)** Percent inhibition of tail currents during the step repolarization protocol, which were calculated from the values in B (* = $P < 0.05$, ** = $P < 0.01$; one-way ANOVAs). n_{WT} = 14, and $n_{S624A,Y652A}$ = 9.
