# Supplementary material for: The molecular determinants of R-roscovitine block of hERG channels
Source: PLoS One. 2019 Sep 3;14(9):e0217733. doi: 10.1371/journal.pone.0217733 (PMC6719874; doi:10.1371/journal.pone.0217733)
Supplement: S2 Fig — (PDF) [file pone.0217733.s002.pdf]

A

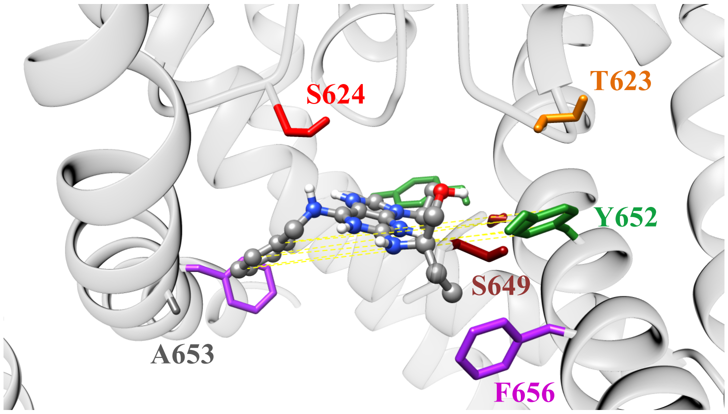

B

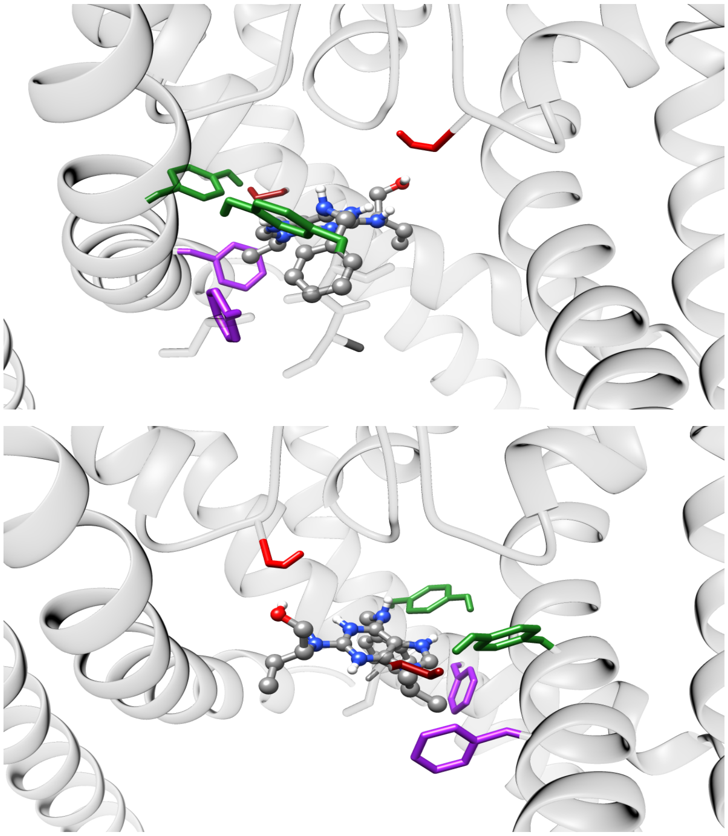

Supplementary Figure 2. Conformations #1 and #4 of *R*-roscovitine docked into an open hERG channel. A) Conformation #1 from Table 1 is shown, with all interacting residues indicated with sticks. *R*-roscovitine is shown in ball & stick. Subunit D was removed for clarity, except S649 from this subunit (dark red). The π-π interaction with Y652 is shown in yellow dotted lines. **B)** (*top*) Conformation #4 from Table 1, shown in a view similar to that in A. Subunit D was removed except for A653 (gray), F656 (green) and Y652 (purple). (*bottom*) A different view of conformation #4 where subunit C was removed, except S649.
