# Supplementary material for: The molecular determinants of R-roscovitine block of hERG channels
Source: PLoS One. 2019 Sep 3;14(9):e0217733. doi: 10.1371/journal.pone.0217733 (PMC6719874; doi:10.1371/journal.pone.0217733)
Supplement: S3 Fig — (PDF) [file pone.0217733.s003.pdf]

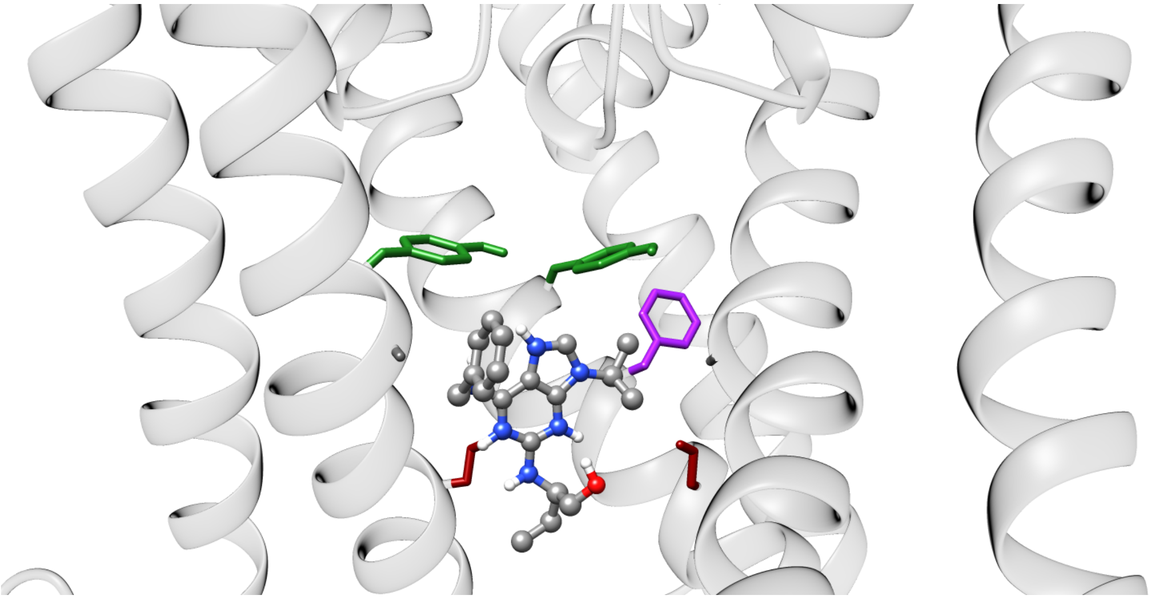

Supplementary Figure 3. *R*-roscovitine docking in the cryo-EM structure. *R*-roscovitine was docked into the pore region of the hERG cryo-EM structure [5] using Autodock Vina [3,4]. Modifications to the structure were made using Chimera [6]. No residues that are adjacent to or in the selectivity filter interacted with *R*-roscovitine (e.g. T623 or S624). Subunit A was removed for clarity. See text for discussion.

References

[1] Farid R, Day T, Friesner RA, Pearlstein RA. New insights about HERG blockade obtained from protein modeling, potential energy mapping, and docking studies. Bioorg Med Chem 2006;14:3160–73. doi:10.1016/j.bmc.2005.12.032.

[2] Jiang Y, Lee A, Chen J, Ruta V, Cadene M, Chait BT, et al. X-ray structure of a voltage-dependent K+ channel. Nature 2003;423:33–41. doi:10.1038/nature01580.

[3] Forli S, Olson AJ. A Force Field with Discrete Displaceable Waters and Desolvation Entropy for Hydrated Ligand Docking. J Med Chem 2012;55:623–38. doi:10.1021/jm2005145.

[4] Morris GM, Huey R, Lindstrom W, Sanner MF, Belew RK, Goodsell DS, et al. AutoDock4 and AutoDockTools4: Automated Docking with Selective Receptor Flexibility. J Comput Chem 2009;30:2785–91. doi:10.1002/jcc.21256.

[5] Wang W, MacKinnon R. Cryo-EM Structure of the Open Human Ether-à-go-go-Related K(+) Channel hERG. Cell 2017;169:422-430.e10. doi:10.1016/j.cell.2017.03.048.

[6] Pettersen EF, Goddard TD, Huang CC, Couch GS, Greenblatt DM, Meng EC, et al. UCSF Chimera--a visualization system for exploratory research and analysis. J Comput Chem 2004;25:1605–12. doi:10.1002/jcc.20084.
