# Supplementary material for: The molecular determinants of R-roscovitine block of hERG channels
Source: PLoS One. 2019 Sep 3;14(9):e0217733. doi: 10.1371/journal.pone.0217733 (PMC6719874; doi:10.1371/journal.pone.0217733)
Supplement: S1 Table — (PDF) [file pone.0217733.s004.pdf]

Subunit providing the interacting residues	Six lowest energy conformations identified					
	#1	#2	#3	#4	#5	#6
A	T623<br>S649<br>Y652(π-π)<br>F656	Y652	S624<br>S649	S624	Y652<br>A653<br>F656	**T623(H-bond)**<br>**S649**
B	S624<br>A653<br>F656	S624<br>S649	Y652	Y652<br>F656	S649<br>Y652	**Y652**<br>**A653**<br>**F656**
C	Y652	S624 (H-bond)<br>A653<br>F656	T623<br>S649<br>F656	S649		**Y652**<br>**F656**
D	S649	T623<br>S649<br>F656	S624<br>A653<br>F656	A653<br>Y652<br>F656	Y652<br>F656	
Kcal/mol	-7.1	-7.1	-7.1	-7.1	-7.1	**-7.1**

Supplementary Table 1. Six lowest energy conformations identified from *R*-roscovitine docking. *R*-roscovitine was docked to a hERG homology model [1] based on the KvAP crystal structure [2], using Autodock Vina [3,4]. Conformations #5 and #6 provided residues that interacted with *R*-roscovitine with three of the four hERG subunits, and conformations #1-4 interacted with all 4 subunits. Hydrogen-bonding was detected in only two conformations: with S624 in conf. #2 and with T623 in conf. #6. The latter was deemed the most likely conformation based on experimental data, and is shown in the paper (Fig 10). A π-π interaction with Y652 was detected in conformation #1, which is shown in supplementary figure 2 below. Conformations #2 and #3 were not deemed as likely because of the extensive involvement of S624, whose mutation does not weaken *R*-roscovitine inhibition. Conformation #4 is shown below in Supplementary Figure 2. Conformation #5 is deemed unlikely because it does not include T623, whose mutation significantly disrupts inhibition; this conformation is also unlikely due to the extensive involvement of Y652, whose mutation causes only a ~2.5 increase in *R*-roscovitine IC_{50}.

References

[1] Farid R, Day T, Friesner RA, Pearlstein RA. New insights about HERG blockade obtained from protein modeling, potential energy mapping, and docking studies. Bioorg Med Chem 2006;14:3160–73. doi:10.1016/j.bmc.2005.12.032.

[2] Jiang Y, Lee A, Chen J, Ruta V, Cadene M, Chait BT, et al. X-ray structure of a voltage-dependent K+ channel. Nature 2003;423:33–41. doi:10.1038/nature01580.

[3] Forli S, Olson AJ. A Force Field with Discrete Displaceable Waters and Desolvation Entropy for Hydrated Ligand Docking. J Med Chem 2012;55:623–38. doi:10.1021/jm2005145.

[4] Morris GM, Huey R, Lindstrom W, Sanner MF, Belew RK, Goodsell DS, et al. AutoDock4 and AutoDockTools4: Automated Docking with Selective Receptor Flexibility. J Comput Chem 2009;30:2785–91. doi:10.1002/jcc.21256.
